# Nanosecond Structure of Radical Pair Intermediates from High-Frequency Quantum Oscillations: Insight into the Q_A_^•–^ to Q_B_ Electron Transfer Step in Purple Bacterial Photosynthesis

**DOI:** 10.1021/acs.jpcb.5c08416

**Published:** 2026-05-20

**Authors:** Hideto Matsuoka, Gerhard Link, Ulrich Heinen, Oleg G. Poluektov, Lisa M. Utschig, Marion C. Thurnauer, Stefan Weber, Gerd Kothe

**Affiliations:** † Division of Chemistry, Hokkaido University of Education, 1-15-55 Shiroyama, Kushiro-shi, Hokkaido 085-0826, Japan; ‡ Institut für Physikalische Chemie, Albert-Ludwigs-Universität Freiburg, Albertstrasse 21, 79104 Freiburg, Germany; § Department of Engineering, Pforzheim University, Tiefenbronner Strasse 65, 75175 Pforzheim, Germany; ∥ Chemical Sciences and Engineering Division, 1291 Argonne National Laboratory, Lemont, Illinois 60439, United States

## Abstract

We demonstrate the validity of our approach to deduce, from the anisotropy of quantum oscillations, the geometry of short-lived radical pair intermediates in photosynthesis. A global fit of a two-dimensional W-band (94 GHz) electron paramagnetic resonance (EPR) experiment provides the same global minimum values for the geometry of the A-side radical pair P_700_^•+^A_1A_^•–^ in photosystem I (PSI) as observed in a previous Q-band (34 GHz) EPR study, yet with a significantly increased convergence rate of 62%. This demonstrates that the global fit yields the correct radical pair geometry even at Q-band frequencies. With this information, we revisit our previous Q-band study of the cofactor arrangement of P_865_^•+^Q_A_^•–^, the stabilized charge-separated state in purple bacterial reaction centers (RCs). Analysis of calculated two-dimensional data sets of P_865_^•+^Q_A_^•–^ reveals that the quantum oscillation technique is unaffected by a mirror ambiguity in disordered solids and thus can provide unambiguous solutions for all five Euler angles of the radical pair geometry. This enables us to elucidate the Q_A_^•–^ to Q_B_ electron transfer step in purple bacterial photosynthesis, the subject of controversial discussions for more than 25 years. Our results show that this electron transfer step involves a gating mechanism requiring a 60° rotation of the headgroup of Q_A_^•–^ in its binding pocket.

## Graphical abstract

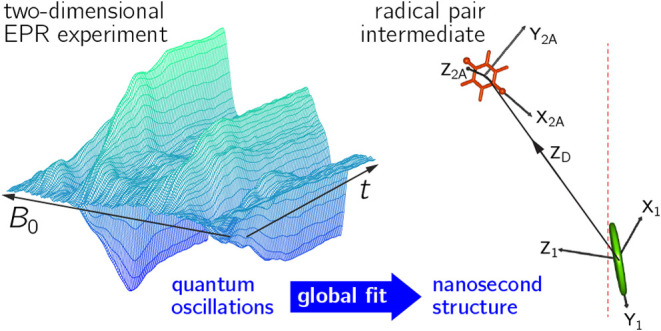

## 1 Introduction

The primary energy conversion steps of photosynthesis proceed via spin-correlated radical pairs as short-lived intermediates.^1^ Charge separation in plant PSI is initiated by photoexcitation of the primary chlorophyll donor, P_700_.^2^ An electron is transported, through an intermediary chlorophyll acceptor A_0_,^2^ from P_700_ in its excited singlet state to a phylloquinone acceptor, A_1_.^3^ The radical pair P_700_^•+^A_1A_^•–^ is the first short-lived intermediate detectable by time-resolved EPR. A_1A_^•–^ denotes A_1_^•–^ on the A branch of the acceptor chains in PSI. At room temperature, P_700_^•+^A_1A_^•–^ decays in about 200 ns by forward electron transfer to the iron–sulfur center F_X_.^4^ At cryogenic temperatures, the lifetime of P_700_^•+^A_1A_^•–^ is about 150 μs, and it decays primarily by charge recombination.^5^ Notably, P_700_^•+^A_1A_^•–^ represents the important energy conversion step of stabilized charge separation across the membrane spanned by the PSI RC protein.

Due to conservation of spin angular momentum in fast light-induced electron transfer, the radical pair state P_700_^•+^A_1A_^•–^ is generated in the same spin multiplicity as its precursor state ^1^P_700_^*^, where ^1^P_700_^*^ is the singlet excited state of P_700_. Generally, such singlet-configured radical pairs are formed with spin-correlated populations of only half of the eigenstates,^6–8^ thus giving rise to high electron spin polarization. In addition, there are zero-quantum electron oscillations^9–17^ and single-quantum nuclear coherences^18^ between the eigenstates of the radical pair, which manifest themselves as quantum oscillations in an EPR experiment conducted with adequate time resolution (Section 1 in Supporting Information (SI)). The zero-quantum electron coherences have been used to explore the three-dimensional structure of radical pair intermediates in the primary energy conversion steps of photosynthesis.^19–25^

Our strategy to obtain the cofactor arrangement of a spin-correlated radical pair involves two-dimensional transient EPR spectroscopy in which zero-quantum and single-quantum electron coherences are correlated with each other. A global fit of this data provides values for the five Euler angles characterizing the radical pair geometry (Section 1 in SI). Generally, the fit is repeated multiple times with different starting values covering the parameter space. The analysis of quantum oscillations from Q-band (34 GHz) transient EPR experiments resulted in about 25% of all runs having the same global minimum values.^23–25^ In the remaining fits different local side minima were reached.

In this study, we first demonstrate the validity of our approach to deduce, from the anisotropy of quantum oscillations, the geometry of short-lived radical pair intermediates in photosynthesis. A computer fit of a two-dimensional W-band (94 GHz) experiment provides the same global minimum values for the geometry of the A-side radical pair P_700_^•+^A_1A_^•–^ in PSI (Section 2 in SI) as observed in a previous Q-band study^25^ yet with a significantly increased convergence rate. This demonstrates that the underlying optimization problem is well-defined and its solution based on an efficient algorithm^26^ yields the correct radical pair geometry even at Q-band frequencies.

Second, with this information we revisit our previous Q-band study in purple photosynthetic bacteria of the cofactor arrangement of P_865_^•+^Q_A_^•–^, the stabilized charge separated state comparable to P_700_^•+^A_1A_^•–^ in PSI.^23^ P_865_ is the primary bacterial chlorophyll donor and Q_A_(Q_B_) is the primary (secondary) ubiquinone acceptor. Analysis of calculated two-dimensional data sets of P_865_^•+^Q_A_^•–^ establishes our previous conjecture of two structurally distinct conformations for Q_A_^•–^.^23,24^ This enables us to elucidate the Q_A_^•–^ to Q_B_ electron transfer step in purple bacterial photosynthesis, the subject of controversial discussions for more than 25 years.

## 2 Methods

### 2.1 Sample Preparation

Lyophilized, whole cells of deuterated (99.7%) cyanobacteria *Synechococcus lividus* were rehydrated in deuterated Tris buffer (uncorrected pH = 7.5) containing 50% glycerol-d_3_ as cryoprotectant. The suspension was then placed in a homemade quartz capillary (0.9 mm o.d. > 0.8 mm i.d.) located in the W-band microwave resonator.

### 2.2 W-band EPR Measurements

The W-band experiments were carried out using a Bruker E600 W-band spectrometer equipped with a TE_011_ cylindrical cavity. The field of the superconducting magnet was calibrated using Li:LiF (Institute of Crystallography, Moscow) as a standard. In the quantum oscillation experiments, performed on frozen aqueous samples, a W-band resonator with a loaded *Q* of 600 was employed. From pulsed electron–electron double resonance (PELDOR) detected nuclear magnetic resonance (NMR) experiments, carried out with a laboratory-made microwave bridge (Krymov bridge), a resonator bandwidth of Δυ ≥ 150 MHz (fwhm) was estimated (data not shown). To improve the sensitivity of our detection system, a two-stage super low-noise preamplifier (SA-230 F5, 400 Hz – 140 MHz) (NF Corporation, Japan) with a final gain of 92 dB was installed into the commercial Bruker W-band bridge. The time-resolved EPR signal was recorded using a Tektronix DPO 3034 digital oscilloscope with a sampling rate of 1.25 GS/s and a bandwidth of 300 MHz. Data acquisition was controlled by LabVIEW 8.6 software (National Instruments Inc., USA). Typically, 500 transients were accumulated to improve the signal-to-noise ratio.

Optical excitation of the samples was performed with 2.5 ns pulses of a *Q*-switched frequency-doubled Nd:YAG laser (Spectra Physics PRO-270) at a repetition rate of 20 Hz. Excitation of the sample in the resonator was achieved using a fiber-optic light path through the sample rod, as described previously.^27^ To improve the excitation efficiency, the fiber with an o.d. of 0.5 mm was inserted into the capillary. The temperature of the samples was controlled using a helium flow cryostat (Oxford Instruments, models CF 935 and ITC 500). All measurements were performed at 80 K. To avoid magneto-orientation effects,^28,29^ the samples were frozen in liquid nitrogen and then inserted into the cooled W-band resonator.

### 2.3 Computations

A Fortran program package was used to analyze the time-resolved EPR data. The programs calculate EPR time profiles and transient spectra of spin-correlated radical pairs with a spatially fixed geometry. The structural parameters were evaluated using a nonlinear least-squares fit procedure based on the Levenberg–Marquardt algorithm.^26^ Parallel execution was utilized for the powder averaging procedure.

## 3 Results and Discussion

### 3.1 Deducing the Radical Pair Geometry of P_700_^•+^A_1A_^•–^ in PSI from W-band Quantum Oscillations

In this study, we employ the fully deuterated whole cells of wildtype cyanobacterium *Synechocccocus lividus* to obtain the geometry of P_700_^•+^A_1A_^•–^ using a two-dimensional transient nutation experiment performed at W-band microwave frequency (ω/(2π) = 94.055 GHz). Generally, a complete data set consists of transient EPR signals taken at equidistant magnetic field points which extend over the full spectral width. This yields a two-dimensional variation of the signal intensity with respect to both the magnetic field and time axis. Figure 1A depicts a complete transient data set measured at W-band frequency and 80 K. The data were obtained with a microwave magnetic field of *B*_1_ = 0.04 mT. Note that Figure 1A depicts only the first 100 ns of the time evolution of the transverse electron spin magnetization after the laser pulse. Positive signal intensities indicate enhanced absorptive (a) and negative emissive (e) spin polarization. Transient spectra can be extracted from this plot at any fixed time after the laser pulse as slices parallel to the magnetic field axis. Likewise, the time evolution of the transverse electron spin magnetization may be obtained for any given field as a slice along the time axis.

**Figure 1:**
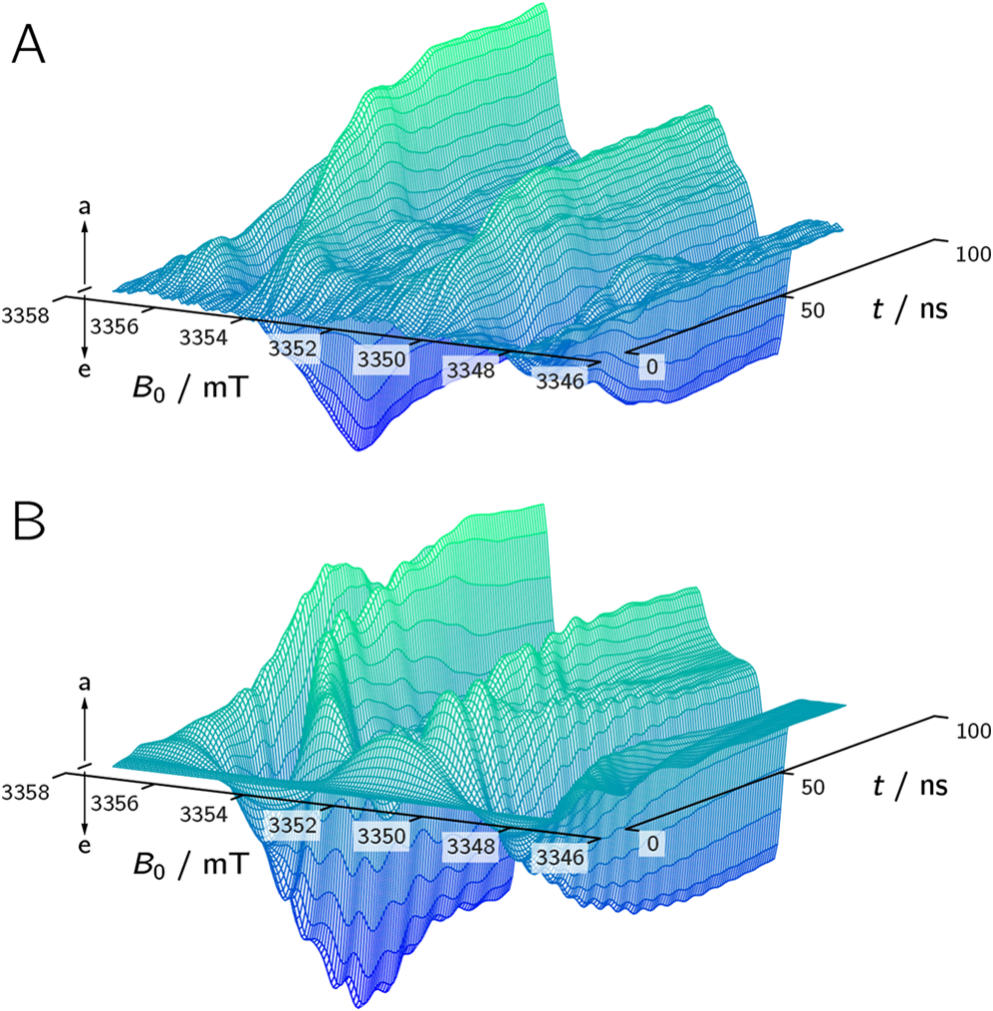
(A) Experimental two-dimensional W-band EPR data set of the A-side radical pair P_700_^•+^A_1A_^•–^ in PSI. Positive signals indicate enhanced absorptive (a) and negative emissive (e) spin polarization. The data set is obtained for fully deuterated whole cells of the cyanobacterium *Synechococcus lividus*, a microwave frequency of ω/(2π) = 94.055 GHz, a microwave field of *B*_1_ = 0.04 mT and *T* = 80 K. (B) Calculated two-dimensional W-band EPR data set for P_700_^•+^A_1A_^•–^ using the structural parameters given in Table 1 (column 2) and the magnetic parameters summarized in Table S1 (Section 3 in SI). A resonator bandwidth of 200 MHz (fwhm) is taken into account by using a Gaussian response function.

Typical W-band EPR spectra, extracted at five different times after the laser pulse, are shown in Figure 2 (solid red lines). Lifetime broadening renders the early spectrum (15 ns) somewhat broader than the later ones.^30^ Moreover, the polarization changes from e/a/e/a/e/a at early times to a characteristic e/a/a/e/a/e/a pattern at later times (75 ns). Figure 3 depicts the short time behavior of the transverse electron spin magnetization (solid red lines) measured at six selected field positions (A–F, Figure 2). Fast initial oscillations are present at early times (up to 100 ns) after the laser pulse. These arise because of zero-quantum electron coherence that is associated with the spin-correlated generation of the radical pair.^9–17^ Note that the amplitude and the frequency ω_ZQ_(Ω) of these coherences vary drastically across the powder spectrum. Study reveals that ω_ZQ_(Ω) covers an exceptionally broad frequency range extending from ω_ZQ_(Ω)/(2π) = 5 MHz to ω_ZQ_(Ω)/(2π) = 230 MHz.

**Figure 2:**
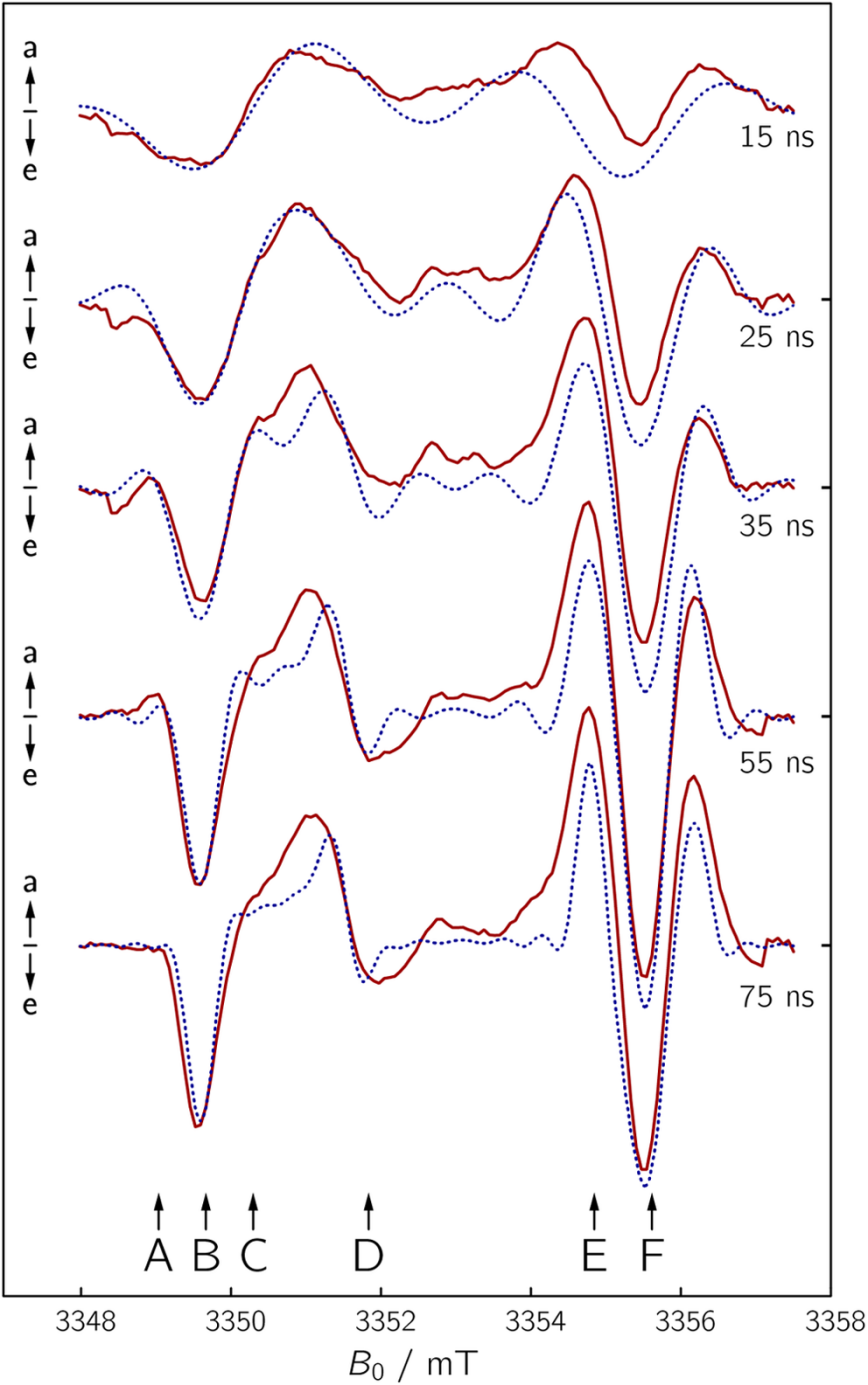
Transient W-band EPR spectra of the A-side radical pair P_700_^•+^A_1A_^•–^ in PSI at various times after the laser pulse. Positive signals indicate enhanced absorptive (a) and negative emissive (e) spin polarization. The spectra are obtained with a microwave frequency of ω/(2π) = 94.055 GHz and a microwave field of *B*_1_ = 0.04 mT. Full red lines: Experimental spectra from fully deuterated whole cells of the cyanobacterium *Synechococcus lividus* at *T* = 80 K. Dotted blue lines: Calculated spectra using the Euler angles given in Table 1 (column 2) and the magnetic parameters listed in Table S1 (Section 3 in SI). A resonator bandwidth of 200 MHz (fwhm) is taken into account by using a Gaussian response function. Time evolutions of the transverse electron spin magnetization at the field positions A to F are shown in Figure 3.

**Figure 3:**
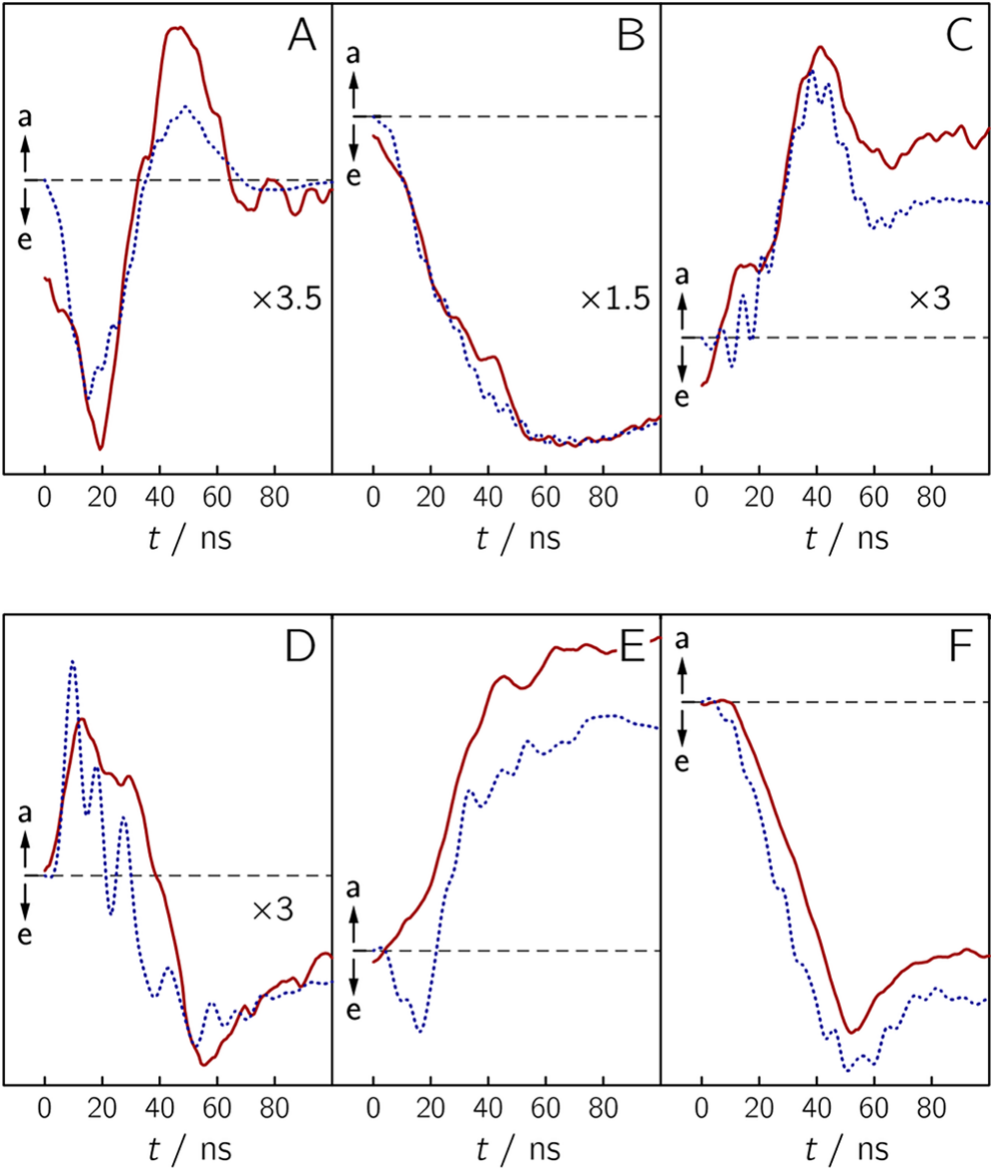
W-band quantum oscillations in the transverse electron spin magnetization of the A-side radical pair P_700_^•+^A_1A_^•–^ in PSI initiated by the laser pulse (*t* = 0). Positive and negative signals indicate enhanced absorptive (a) and emissive (e) spin polarizations, respectively. The transients refer to a microwave frequency of ω/(2π) = 94.055 GHz, a microwave field of *B*_1_ = 0.04 mT and six different magnetic fields (positions A–F, Figure 2). Full red lines: Experimental time profiles from fully deuterated whole cells of the cyanobacterium *Synechococcus lividus* at *T* = 80 K. Dotted blue lines: Calculated time profiles using the Euler angles given in Table 1 (column 2) and the magnetic parameters summarized in Table S1 (Section 3 in SI). A resonator bandwidth of 200 MHz (fwhm) is taken into account by using a Gaussian response function.

The orientation of the magnetic tensors of P_700_^•+^A_1A_^•–^ with respect to a magnetic reference system is described by the five Euler angles Φ_1A_, Θ_1A_, Ψ_1A_, Θ_DA_ and Ψ_DA_. They constitute the geometry of the radical pair (Section 1 in SI). Values for these angles were obtained from a global fit of the two-dimensional W-band EPR data using the fixed magnetic parameters summarized in Table S1 (Section 3 in SI). Notably, the full spectral width of 9.52 mT and a time range of 99.2 ns (Δ*t* = 1.6 ns) after the laser pulse were considered. Thus, 69 calculated time profiles, involving 4347 data points, were fitted to the experimental profiles by varying the Euler angles of the radical pair geometry. Repetitive fittings with different starting values were performed to cover the parameter space. In a striking 62% of all runs the same global minimum values were found. Local side minima were reached in the remaining fits. In the Figures 1, 2 and 3, we compare the complete experimental data set as well as selected line shapes and time profiles with the best fit simulation based on the angular values listed in Table 1 (column 2). The cited errors are systematic errors resulting from uncertainties in the published **g** tensor components.^23,25^

**Table 1:** Comparison of W- and Q-Band Geometries of the A-Side Radical Pair ***P***_**700**_^•**+**^ ***A***_**1** *A*_^•**‑**^ **in PSI**

W-band geometry^a^				Q-band geometry^b^		
notation^c^	angular value	error^d^	convergence rate	angular value	error^d^	convergence rate
Φ_1A_	228°	±4°		226°	±4°	
Θ_1A_	95°	±13°		88°	±13°	
Ψ_1A_	330°	±3°	62%	319°	±3°	22%
Θ_DA_	78°	±3°		71°	±3°	
Ψ_DA_	15°	±6°		11°	±6°	

a Evaluated in the present study using a global fit of a two-dimensional W-band EPR data set.

b Adopted from a two-dimensional Q-band EPR study.^25^

c The Euler angles Φ_1A_, Θ_1A_, Ψ_1A_, Θ_DA_ and Ψ_DA_ relate the principal axis system of the respective magnetic tensor and the magnetic reference system.

d The cited errors consider the uncertainties in the published **g** tensor components.^23,25^

Generally, the agreements between the experimental data and the calculations are good. We suggest that the deviations observed (Figures 1–3) are due to neglect in the calculations of sequential electron transfer polarization (SETP)^31–33^ between the primary and secondary radical pairs, P_700_^•+^A_0A_^•–^ and P_700_^•+^A_1A_^•–^, respectively. In the SETP mechanism, electron spin polarization developed in P_700_^•+^A_0A_^•–^ is projected onto the polarization developed in P_700_^•+^A_1A_^•–^. The magnitudes of the effects of SETP are dependent on the lifetime of the primary radical pair, P_700_^•+^A_0A_^•–^,^31–33^ and the strength of the external magnetic field.^34^ We expect even with a lifetime as short as 50 ps, the SETP effects to be noticeable because of the high magnetic field (3.35 T ≤ *B*_0_ ≤ 3.36 T) employed in our W-band study. It is noteworthy that in a previous Q-band study of the radical pair P_865_^•+^Q_A_^•–^ ^23^ with 1.213 T ≤ *B*_0_ ≤ 1.216 T, the divergences are markedly smaller (Section 6 in SI).

A general problem in the analysis of magnetic resonance experiments is degeneracy since these techniques cannot in general distinguish between a positive and a negative magnetic axis orientation (Section 4 in SI).^23,25^ As a consequence, the given geometry represents a selected set of 32 magnetically equivalent tensor orientations obtained from the fit of the two-dimensional W-band data. The 32 equivalent geometries can be divided into eight groups with four geometries where the latter yield indistinguishable spatial structures.Thus, only an 8-fold degeneracy exists.

Previously, we have studied the electron transfer pathways in PSI (Section 2 in SI) by high-time resolution Q-band EPR in the deuterated green alga *Chlamydomonas reinhardtii* at cryogenic temperature.^25^ From a two-dimensional Q-band experiment, performed on wildtype cells, we extracted a mole ratio of 0.71/0.29 in favor of the A-side radical pair P_700_^•+^A_1A_^•–^ with the selected geometry listed in Table 1 (column 5). The convergence rate of the fit was 22%. The cited errors are systematic errors attributed to the uncertainties in the published **g** tensor components.^23,25^ Inspection of Table 1 reveals that the W- and Q-band geometries of P_700_^•+^A_1A_^•–^ are identical within the error limits. Yet, the convergence rate of the W-band fit is 62%, three times larger than that of the Q-band fit.

### 3.2 Evaluating the Three-dimensional Structure of P_700_^•+^A_1A_^•–^ Using W-Band Quantum Oscillations and High-Time Resolution Multi-Frequency EPR

To determine the three-dimensional structure of P_700_^•+^A_1A_^•–^ in PSI, knowledge of the orientation of one **g** tensor in an external reference system is required. This information is available for P_700_^•+^ in the A-side radical pair, for which the **g** tensor was determined using high-time resolution X-(9.5 GHz) and W-band EPR on deuterated and ^15^N-substituted cyanobacteria.^20^ To describe the **g** tensor orientation of P_700_^•+^ in P_700_^•+^A_1A_^•–^, we employ a chlorophyll-based reference system which corresponds to the reference system used for the primary donor in purple bacteria.^35^ In this reference system, the orientation of the **g** tensor of P_700_^•+^ in P_700_^•+^A_1A_^•–^ can be described by four equivalent sets of Euler angles (Table S3, Section 5 in SI).^25^

A critical evaluation of the radical pair structures calculated from the eight degenerate W-band geometries and the four possible **g** tensor orientations allows for the removal of the degeneracy. The crucial parameter is the position of A_1A_^•–^ in the photosynthetic membrane. It is assumed that a shift of A_1A_^•–^ relative to the position of A_1A_ in the X-ray structure beyond the EPR uncertainty of ± 5.5 Å is unlikely to occur. One can eliminate seven of the eight W-band geometries and three of the four **g** tensor orientations using the quinone position as selection criterion. This results in a unique set of Euler angles:

$$\begin{array}{l} \begin{array}{ll} \Phi_{1A}=228^{{}^{\circ}} & \Phi_{1}^{\mathrm{Chl}}=183^{{}^{\circ}}\\ \Theta_{1A}=95^{{}^{\circ}} & \Theta_{1}^{\mathrm{Chl}}=29^{{}^{\circ}}\\ \Psi_{1A}=330^{{}^{\circ}} & \Psi_{1}^{\mathrm{Chl}}=220^{{}^{\circ}}\\ \Theta_{\mathrm{DA}}=78^{{}^{\circ}} & \\ \Psi_{\mathrm{DA}}=15^{{}^{\circ}} & \\ selected\;W‐band\;geometry\,\;of{\;P}_{700}^{•+}A_{1A}^{•-} & orientation\;I\;of\;\,\mathrm{the}\,\;g\;tensor\;of\;P_{700}^{•+}\;in\;P_{700}^{•+}A_{1A}^{•-} \end{array} \end{array}$$

Figure 4A shows the three-dimensional structure of the A-side radical pair P_700_^•+^A_1A_^•–^ in PSI. The EPR model is based on the point-dipole approximation for the electron dipolar interactions. We assume that the chlorophyll *a* species, coordinated by the PsaB protein subunit, carries the major part of the electron spin in the primary donor.^36^ Figure 4B depicts the cofactor arrangement of P_700_A_1A_ as determined by X-ray crystallography.^2^ Comparison with Figure 4A reveals that the orientation and position of the quinone headgroup of A_1A_ in both structures are very similar. We therefore conclude that our technique provides the correct radical pair geometry underlying the X-ray structure.

**Figure 4:**
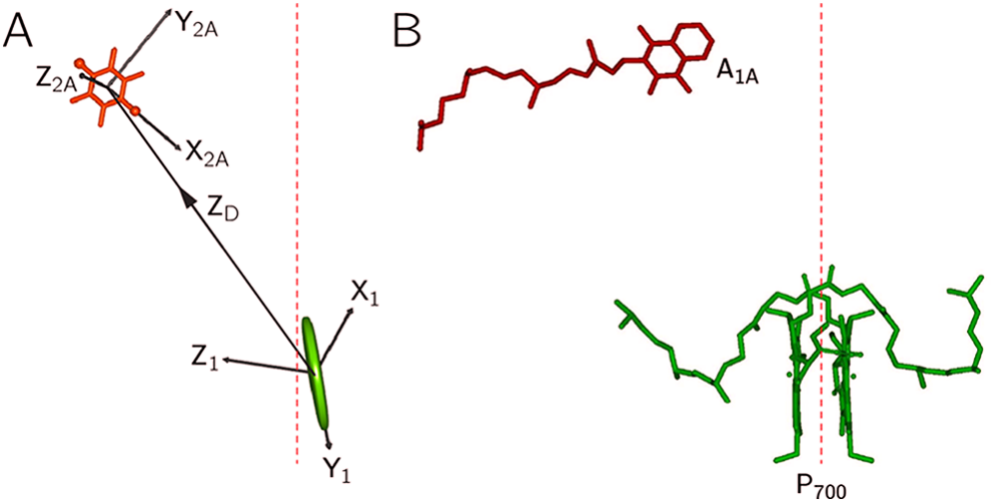
(A) Three-dimensional structure of the A-side radical pair P_700_^•+^A_1A_^•–^ in PSI determined by W-band quantum oscillations (present study) in combination with high-time resolution X- and W-band EPR.^20^ The direction view is parallel to the membrane plane. The green disk represents chlorophyll *a* in the primary donor P_700_ coordinated by the PsaB protein subunit carrying the major part of the unpaired electron spin.^36^ ***X***_1_, ***Y***_1_, ***Z***_1_: principal axis system of the **g** tensor of P_700_^•+^. ***X***_2A_, ***Y***_2A_, ***Z***_2A_: principal axis system of the **g** tensor of A_1A_^•–^. ***Z***_D_: symmetry axis of the dipolar tensor. (B) Cofactor arrangement of the primary donor P_700_ and the A-side quinone acceptor A_1A_ (a phylloquinone) in PSI determined by X-ray crystallography.^2^ The dashed red lines indicate the approximate *C*_2_ symmetry axis collinear with the membrane normal.

Summarizing the efficacy of the quantum oscillation technique, we want to make two points: First, the above results show that the underlying optimization problem is numerically well-defined and its solution based on the Levenberg–Marquardt algorithm^26^ provides the correct radical pair geometry even at Q-band frequencies. Second, a particular set of Euler angles, obtained from the global fit, always represents only one of 32 equivalent geometries connected by 32 given sets of magnetic tensor rotations (Table S2, Section 4 in SI).^23,25^ Consequently, if two geometries cannot be transformed into each other by any of these tensor rotations, these are incompatible geometries corresponding to two distinct radical pair structures.

### 3.3 Detecting Two Structurally Distinct Conformations for Q_A_ in the RCs of Purple Bacteria

In a previous Q-band EPR study of the radical pair P_865_^•+^Q_A_^•–^(Section 6 in SI)^23^ in fully deuterated Fe-removed/Zn-substituted RCs (Section 7 in SI)^37,38^ of the purple bacterium *Rhodobacter sphaeroides*, we observed that the geometries of P_865_^•+^Q_A_^•–^, extracted from the two-dimensional Q-band data set and the X-ray structure of the RC protein,^39^ were not compatible with each other, i.e.,:

$$\mathrm{EPR},\begin{array}{l} \Phi_{1}=26^{{}^{\circ}}\pm 4^{{}^{\circ}}\\ \Theta_{1}=114^{{}^{\circ}}\pm 8^{{}^{\circ}}\\ \Psi_{1}=73^{{}^{\circ}}\pm 5^{{}^{\circ}}\\ \Theta_{D}=67^{{}^{\circ}}\pm 3^{{}^{\circ}}\\ \Psi_{D}=122^{{}^{\circ}}\pm 2^{{}^{\circ}} \end{array},X‐\mathrm{ray},\begin{array}{l} \begin{array}{l} \Phi_{1}=19^{{}^{\circ}}\pm 8^{{}^{\circ}}\\ \Theta_{1}=120^{{}^{\circ}}\pm 12^{{}^{\circ}}\\ \Psi_{1}=16^{{}^{\circ}}\pm 10^{{}^{\circ}}\\ \Theta_{D}=73^{{}^{\circ}}\pm 1^{{}^{\circ}}\\ \Psi_{D}=68^{{}^{\circ}}\pm 4^{{}^{\circ}} \end{array} \end{array}$$

The cited errors in the EPR geometry are systematic errors based on the uncertainties in the published **g** tensor components,^23^ whereas the errors in the X-ray geometry are derived from the variation of the angular values in five different X-ray structures of the same RC protein.^23^ Comparison reveals that three out of the five Euler angles agree within the error limits. Only the angles Ψ_1_ and Ψ_D_ deviate by about 60° between the two geometries. This raises an interesting question which is connected with a mirror ambiguity of EPR solutions in disordered solids^40^ (Supporting Information of ref^40^). Can the quantum oscillation technique distinguish between the two geometries?

To answer this question, we calculated two-dimensional Q-band EPR data sets for both options. The underlying magnetic and structural parameters are listed in Table S4 (Section 8 in SI). Figure 5 depicts the time evolution of the transverse electron spin magnetization, extracted at six selected field positions (A – F) from the two-dimensional data sets. Here red solid lines refer to the EPR geometry whereas blue solid lines indicate the X-ray geometry. Inspection reveals that in the P_865_^•+^ spectral range (D – F), the modulations in the transverse electron spin magnetization are rather similar. On the other hand, within the magnetic field range in which the Q_A_^•–^ resonances are dominant (A – C), the amplitudes of the quantum oscillations associated with the two geometries differ markedly from each other.

**Figure 5:**
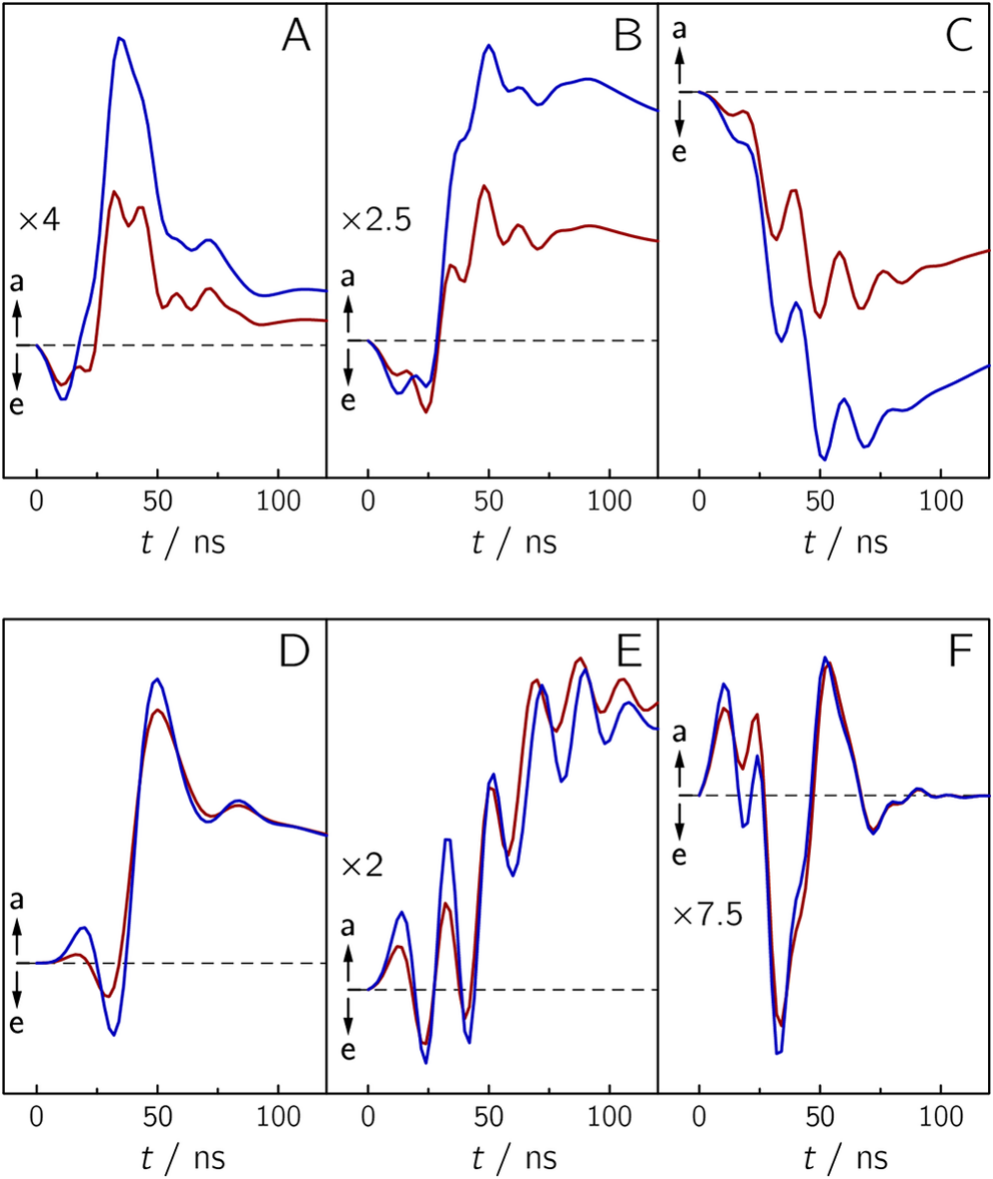
Calculated Q-band quantum oscillations of the radical pair P_865_^•+^Q_A_^•–^ in RCs of purple bacteria for two different geometries using the magnetic and structural parameters in Table S4 (Section 8 in SI). Positive and negative signals indicate enhanced absorptive (a) and emissive (e) spin polarizations, respectively. The transients refer to a microwave frequency of ω/(2π) = 34.064 GHz, a microwave field of *B*_1_ = 0.04 mT and six different static magnetic fields, i.e., (A) *B*_0_ = 1212.671 mT, (B) *B*_0_ = 1212.871 mT, (C) *B*_0_ = 1213.423 mT, (D) *B*_0_ = 1214.726 mT, (E) *B*_0_ = 1215.578 mT, and (F) *B*_0_ = 1216.429 mT. A resonator bandwidth of 110 MHz (fwhm) is taken into account by using a Gaussian response function. Full red lines: Time profiles calculated for the EPR geometry. Full blue lines: Time profiles calculated for the X-ray geometry.

To support this result, each of the two calculated data sets was fitted to the experimental data set by varying two scale factors (amplitude and frequency). From the sum of squared errors of these fits, we then determined the *R*-factor of the two geometries according to

$$R\left(k\right)=\sqrt{\underset{i}{\sum }{\left(x_{i}-\xi_{\mathrm{ik}}\right)}^{2}/\underset{i}{\sum }x_{i}^{2}}$$ 1

Here *x*_*i*_ denotes the measured amplitudes taken from ref 23, ξ_*ik*_ are the calculated amplitudes and *k* stands for EPR and X-ray geometry, respectively. Study reveals noticeably different *R*-factors of *R*(EPR) = 14.9% and *R*(X-ray) = 33.8%. Obviously, the quantum oscillation technique can distinguish between the two geometries. This implies that our method is unaffected by a *mirror ambiguity in disordered solids* and thus can provide *unambiguous solutions for all five Euler angles* of the radical pair geometry.

At this point, it is appropriate to compare our result with that of Savitsky et al.^41^ who employed a multidimensional EPR technique to evaluate the spatial structure of P_865_^•+^Q_A_^•–^ in its native membrane. Performing orientation-resolving high-field PELDOR, the authors argued that, apart from a small light-induced reorientation, the cofactor arrangement of P_865_^•+^Q_A_^•–^ is virtually the same as that of P_865_Q_A_ determined by X-ray crystallography. Their statement was based on the notion that all five Euler angles of the radical pair geometry can definitely be determined by orientation-resolving high-field PELDOR. This is true for a complete three-dimensional PELDOR experiment with independent full-range sweeps of all three primary variables.^40^ However, since such a strategy is very time-consuming, only a simplified two-dimensional PELDOR experiment was performed. As a result, only three of the five Euler angles of the radical pair geometry can clearly be extracted, whereas for the fourth and fifth Euler angles, responsible for the distinction between EPR and X-ray geometry, an ambiguity remained^40^ (Supporting Information of ref^40^).

For three-dimensional structure determination of P_865_^•+^Q_A_^•–^ in the photosynthetic membrane, it is essential to know the orientation of one **g** tensor in an external reference system. This information exists for P_865_^•+^ where the **g** tensor was determined by single crystal W-band EPR.^35^ Using a reference system, which reflects the local symmetry of the bacteriochlorophyll dimer, the orientation of the **g** tensor of P_865_^•+^ has been described by four equivalent sets of Euler angles.^23^ This degeneracy can be removed by a critical evaluation of the radical pair structures calculated from the eight degenerate EPR geometries and the four possible **g** tensor orientations. Using the quinone position as selection criterion, a unique set of Euler angles is obtained. The three-dimensional structure of P_865_^•+^Q_A_^•–^ in the photosynthetic membrane a few tens of nanoseconds after light-induced charge separation is shown in Figure 6A. Comparison with data from X-ray crystallography^39^ reveals that the position of the quinone is essentially the same (Figure 6B). However, the headgroup of Q_A_^•–^ is rotated by about 60° in the ring plane relative to its orientation in the crystal structure. Clearly, this study reveals two structurally distinct states of Q_A_ in the RCs of purple bacteria, as conjectured previously.^23,24^

**Figure 6:**
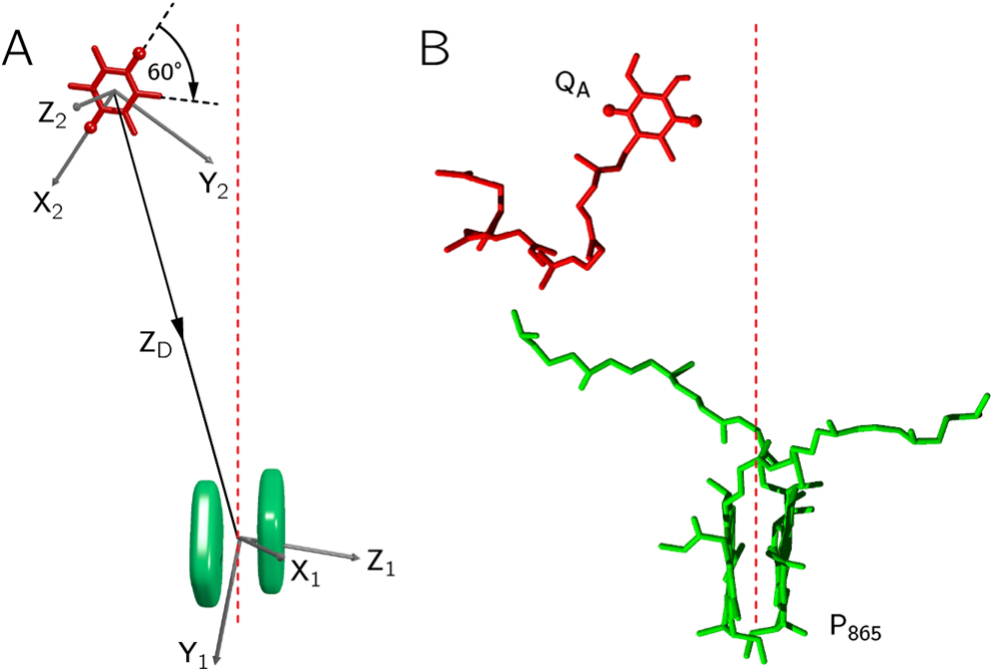
(A) Three-dimensional structure of the radical pair P_865_^•+^Q_A_^•–^ in RCs of purple bacteria determined by Q-band quantum oscillations^23^ in combination with single crystal W-band EPR.^35^ The direction view is parallel to the membrane plane. The green disks are the bacteriochlorophyll molecules of the primary donor P_865_. ***X***_1_, ***Y***_1_, ***Z***_1_: principal axis system of the **g** tensor of P_865_^•+^. ***X***_2_, ***Y***_2_, ***Z***_2_: principal axis system of the **g** tensor of Q_A_^•–^. ***Z***_D_: symmetry axis of the dipolar tensor. The EPR structure represents the cofactor arrangement of the radical pair P_865_^•+^Q_A_^•–^(EPR) where Q_A_^•–^(EPR) is the spatial arrangement of the neutral Q_A_ in the dark adapted RC protein. (B) Cofactor arrangement of the primary donor P_865_ and the primary quinone acceptor Q_A_ determined by X-ray crystallography.^39^ Because of radiation damage,^43^ the X-ray structure displays the spatial arrangement of the reduced quinone Q_A_^•–^(X-ray). Dashed red lines indicate the approximate local *C*_2_ axis of the bacteriochlorophyll dimer.

In the two Q_A_ conformations, the spatial orientation of the carbonyl bonds is different. This might be relevant to the rate of electron transfer from the reduced intervening bacteriopheophytin acceptor, Φ_A_^•–^, to Q_A_ and from Q_A_^•–^ to the secondary quinone acceptor, Q_B_. To check this intriguing notion, we first examine Q_A_^•–^(EPR)­(Figure 6A) which is obtained at 70 K a few tens of nanoseconds after light-induced charge separation. We identify Q_A_^•–^(EPR) with the arrangement of Q_A_ prior to the electron transfer from Φ_A_^•–^, since large-scale motions of Q_A_^•–^(EPR) are unlikely to occur under these conditions. This conformation thus represents the spatial arrangement of the neutral Q_A_ in the dark adapted RC protein.

Let us now examine Q_A_(X-ray)­(Figure 6B). Comparison reveals that the quinone is rotated by 60° relative to the EPR conformation. How do we explain this structural change? One plausible explanation involves the following. It is well-known that X-ray crystallography of protein crystals causes a certain amount of specific radiation damage, i.e., chemistry occurring as a function of X-ray dose.^42^ Generally, radiation-induced photoelectrons are trapped on specific susceptible protein groups initiating structural and chemical alterations, including breakage of disulfide bonds and decarboxylation.^42^ In the case of photosynthetic RC protein crystals the ‘damage/radiation chemistry’ can result in electrons trapped on the RC cofactors. Particularly relevant to the present results is a test study of radiation damage in single crystals of Fe-removed and Zn-substituted RCs of *Rhodobacter sphaeroides*:^43^ low doses of irradiation caused the quinones to be reduced and led to a loss of photosynthetic activity.^43^ Thus, we identify Q_A_(X-ray) with the spatial arrangement of the reduced quinone Q_A_^•–^(X-ray) which differs from that of Q_A_^•–^(EPR) by a 60° rotation of the headgroup.

### 3.4 Elucidating the Q_A_^•–^ **to** Q_B_ Electron Transfer Step in Purple Bacterial Photosynthesis

The rate of electron transfer from Q_A_^•–^ to Q_B_, *k*_AB_, was measured in RCs of *Rhodobacter sphaeroides* in which the native ubiquinone in the Q_A_ binding site was replaced with quinones having different redox potentials.^44^ The results showed that *k*_AB_ does not change as the redox free energy for electron transfer is varied. It was therefore concluded that the Q_A_^•–^→ Q_B_ electron transfer is conformationally gated, i.e., that the rate-limiting step is a conformational change required before electron transfer.^44^ This change was proposed to be a movement of Q_B_ as observed in an X-ray study of the RC protein, frozen under illumination and in the dark.^45^ However, Fourier transform infrared studies of RCs of *Rhodobacter sphaeroides* suggest that this movement of Q_B_ is not related to the Q_A_^•–^→ Q_B_ electron transfer.^46^

Having established two structurally distinct conformations for Q_A_^•–^, we are able to elucidate the Q_A_^•–^ to Q_B_ electron transfer step in purple bacterial photosynthesis.^23,24^ Thus, the formation of P_865_^•+^Q_B_^•–^ involves four consecutive steps, which are depicted in part in Figure 7

$$P_{865}\Phi_{A}Q_{A}\left(\mathrm{EPR}\right)Q_{B}\overset{h\nu ,200\mathrm{ps}}{\to }P_{865}^{•+}\Phi_{A}Q_{A}^{•-}(\mathrm{EPR})Q_{B}\overset{k_{C}\;,{\left(100\mathrm{\mu s}\right)}^{-1}\;}{\to }P_{865}^{•+}\Phi_{A}Q_{A}^{•-}\left(X‐\mathrm{ray}\right)Q_{B}\overset{k_{\mathrm{ET}}\;\gg k_{C}}{\to }P_{865}^{•+}\Phi_{A}Q_{A}(X‐\mathrm{ray})Q_{B}^{•-}\overset{k_{R}}{\to }P_{865}^{•+}\Phi_{A}Q_{A}(\mathrm{EPR})Q_{B}^{•-}$$

Charge separation in the RCs of *Rhodobacter sphaeroides* is initiated by photoexcitation of the primary bacteriochlorophyll donor, P_865_.^47^ Within 200 ps (295 K), an electron is transferred from P_865_ in its excited singlet state to the primary quinone acceptor, Q_A_,^48^ via the intervening bacteriopheophytin acceptor,Φ_A_(Figure 7A).^47^ Using the quantum oscillation technique, we obtain the three-dimensional structure of the radical pair P_865_^•+^Q_A_^•–^(EPR) a few tens of nanoseconds after light-induced charge separation where Q_A_^•–^(EPR) is the inactive conformation (Figure 6A). Conversion to the active conformation Q_A_^•–^(X-ray) (Figure 6B) occurs with the conformational gating rate *k*_C_ = *k*_AB_ = (100 μs)^−1^ (296 K).^44^ The gating step involves a rotation of the headgroup of Q_A_^•–^(EPR) in its binding pocket by 60° (Figure 7B). Once Q_A_^•–^ assumes its active configuration, the electron transfer can occur with the rate *k*_ET_ ≫ *k*_C_ (Figure 7C). Subsequent to electron transfer, Q_A_ returns to the EPR conformation, characteristic of the neutral quinone in the dark adapted protein. The rate of this process is *k*_R_

**Figure 7:**
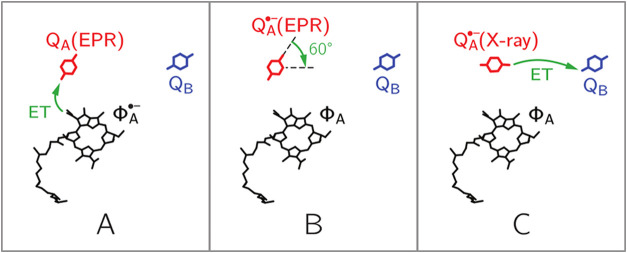
Three-dimensional representation of primary energy conversion steps in RCs of purple bacteria. (A) Electron transfer (ET) from the reduced intermediate acceptor Φ_A_^•–^ to the first quinone acceptor Q_A_(EPR) where Q_A_(EPR) is the inactive conformation which inhibits the electron transfer to the secondary quinone acceptor Q_B_. (B) Gating mechanism effective in the electron transfer from Q_A_^•–^ to Q_B_.The gating step involves a 60° rotation of the headgroup of Q_A_^•–^(EPR)­in its binding pocket (gating rate *k*_C_ = (100 μs)^−1^). (C) Electron transfer from the active conformation Q_A_^•–^(X-ray) to Q_B_(electron transfer rate *k*_ET_ ≫ *k*_C_). Both Q_A_ and Q_B_ are ubiquinones.

The presented gating mechanism is consistent with an observation by Kleinfeld et al.^49^ who reported that electron transfer from Q_A_^•–^to Q_B_ proceeds in RCs cooled to cryogenic temperature under illumination but does not proceed in RCs cooled in the dark.^49^ RCs frozen in the dark are trapped in the inactive conformation Q_A_^•–^(EPR). Obviously, there is insufficient thermal energy for conformational interconversion at cryogenic temperature and consequently electron transfer is inhibited. RCs cooled under illumination are trapped in the active conformation Q_A_^•–^(X-ray) which can undergo electron transfer even at cryogenic temperature. Our mechanism also accounts for another surprising observation made on RCs containing only Q_A_.^49^ When these RCs were cooled in the dark, the charge recombination time of the process P_865_^•+^Q_A_^•–^→P_865_Q_A_was τ_AP_^dark^ ≈ 25 ms (77 K). Charge recombination is significantly prolonged (τ_AP_^light^ ≈ 120 ms (77 K)) when the same RCs were cooled under illumination.^49^ These findings are easily rationalized in terms of the two distinct Q_A_^•–^ conformations depicted in Figure 6.

## 4 Conclusions

The quantum oscillation technique, described in this study, represents an efficient tool that can yield simultaneous structural and mechanistic details on a nanosecond time scale. The potential of this technique is best illustrated by application to study photosynthetic energy conversion in RC proteins. We have presented details of the quantum oscillation technique when executed at high microwave frequencies. A global fit of a two-dimensional W-band EPR experiment provided the same global minimum values for the radical pair geometry of P_700_^•+^A_1A_^•–^ in PSI as observed in a previous Q-band study^25^ yet with a significantly increased convergence rate of 62%. This shows that the underlying optimization problem is numerically well-defined and its solution based on the Levenberg–Marquardt algorithm^26^ yields the correct radical pair geometry even at Q-band frequencies.

With this information, we revisited our previous Q-band study of P_865_^•+^Q_A_^•–^, the initial “stable” charge separated state in purple bacterial RCs. Analysis of calculated two-dimensional data sets of P_865_^•+^Q_A_^•–^ revealed that the quantum oscillation technique is unaffected by a mirror ambiguity in disordered solids and thus can provide unambiguous solutions for all five Euler angles of the radical pair geometry. This enabled us to elucidate the Q_A_^•–^ to Q_B_ electron transfer step in purple bacterial photosynthesis, the subject of controversial discussions for more than 25 years.

Although our study was concentrated on photosynthetic proteins, quantum oscillations of spin-correlated radical pairs are a common feature of systems that undergo efficient light-induced charge separation. Apparently, these shared characteristics of natural^9–15,18,20–25^ and artificial^16,17,19^ photosynthetic systems, as revealed through time-resolved and pulsed EPR, reflect underlying fundamental structural and kinetic requirements for efficient charge separation. These are also expected to be relevant in a number of photoactive flavoproteins. For example, in photolyases and cryptochromes, which are enzymes for light-induced repair of UV-induced DNA lesions and blue-light receptor proteins involved in circadian rhythms, respectively, spin-correlated radical pairs comprising a flavin radical and a tryptophan (tyrosine) radical have been observed using time-resolved variants of EPR.^50,51^ In the cryptochrome subclade, quantum effects were suggested to play a critical role in their proposed function as photomagnetoreceptor in the magnetic compass of migratory birds by virtue of magnetic-field-dependent singlet-to-triplet interconversion of radical pair states.^52^ Thus, observation of quantum oscillations from these proteins may not only provide a crucial test for the radical pair hypothesis of magnetoreception but also may be exploited to study light-induced changes in the geometry of reaction intermediates. We therefore expect that the quantum oscillation technique will continue to yield new insights into the biophysical and molecular mechanisms of proteins that undergo efficient light-induced charge separation.

## Supplementary Material



## Notes

Theoretical background; bidirectional electron transfer in photosystem I; magnetic parameters derived from spectral simulations; information on the degeneracy of the radical pair geometry; transient Q-band EPR data of the radical pair P_865_^•+^Q_A_^•–^ in purple bacteria; Fe-removed/Zn-substituted bacterial reaction centers; magnetic and structural parameters of P_865_^•+^Q_A_^•–^ (PDF)

## Notes

This contribution is dedicated to the memory of Professor Seigo Yamauchi, an outstanding scientist and great friend who died much too early at the age of 64.
