# Supplementary material for: Nanosecond Structure of Radical Pair Intermediates from High-Frequency Quantum Oscillations: Insight into the QA •– to QB Electron Transfer Step in Purple Bacterial Photosynthesis
Source: J Phys Chem B. 2026 May 20;130(22):5450–9. doi: 10.1021/acs.jpcb.5c08416 (PMC13244454; doi:10.1021/acs.jpcb.5c08416)
Supplement: Supplementary file 1 [file jp5c08416_si_001.pdf]

Supporting Information

Nanosecond Structure of Radical Pair Intermediates from High-Frequency Quantum Oscillations: Insight into the $Q_A^{\bullet-}$ to Q_B Electron Transfer Step in Purple Bacterial Photosynthesis

Hideto Matsuoka [a], Gerhard Link [b], Ulrich Heinen [c], Oleg G. Poluektov [d], Lisa M. Utschig [d], Marion C. Thurnauer [d], Stefan Weber [b], and Gerd Kothe [*] [b]

[a] Division of Chemistry, Hokkaido University of Education
Kushiro-shi, Hokkaido 085-0826 (Japan)

[b] Institut für Physikalische Chemie, Albert-Ludwigs-Universität Freiburg
79104 Freiburg (Germany)

[c] Department of Engineering, Pforzheim University
75175 Pforzheim (Germany)

[d] Chemical Sciences and Engineering Division, Argonne National Laboratory
Lemont, IL 60439 (United States of America)

* E-mail: gerd.kothe@physchem.uni-freiburg.de

Table of Contents

1 Theoretical background ……………………………………………………………….. S3

2 Bidirectional electron transfer in photosystem I ……………………………......... S6

3 Magnetic parameters of $P_{700}^{\bullet+}A_{1A}^{\bullet-}$ ……………………………………………………...S7

4 Degeneracy of the radical pair geometry ……………………………………...S9

5 g Tensor orientation of $P_{700}^{\bullet+}$ in $P_{700}^{\bullet+}A_{1A}^{\bullet-}$ ………………………………………....... S11

6 Transient Q-band EPR data of $P_{865}^{\bullet+}Q_{A}^{\bullet-}$ ……………………………………….. S12

7 Fe-removed / Zn-substituted purple bacterial reaction centers ……………………. S13

8 Magnetic and structural parameters of $P_{865}^{\bullet+}Q_{A}^{\bullet-}$ ……………………………………… S13

9 References ……………………………………………………………………….... S15

1 Theoretical background

In this section, we briefly summarize a quantum mechanical model used in the analysis of two-dimensional W-band (94 GHz) electron paramagnetic resonance (EPR) experiments on the radical pair $P_{700}^{\bullet+}A_{1A}^{\bullet-}$ in photosystem I (PSI). Particular emphasis is given to zero-quantum electron oscillations detectable at early times after pulsed laser excitation.[1-9]

Spin Hamiltonian. The spin Hamiltonian employed, H, considers Zeeman, dipolar, exchange, and hyperfine interactions of the radical pair. Thus, H can be written as

$$H = \beta \mathbf{B}_0 \cdot (\mathbf{g}_1 \cdot \mathbf{S}_1 + \mathbf{g}_2 \cdot \mathbf{S}_2) + 2\mathbf{S}_1 \cdot \mathbf{D} \cdot \mathbf{S}_2 - 2J\,\mathbf{S}_1 \cdot \mathbf{S}_2 + \sum_k \mathbf{S}_1 \cdot \mathbf{A}_{1k} \cdot \mathbf{I}_{1k} + \sum_l \mathbf{S}_2 \cdot \mathbf{A}_{2l} \cdot \mathbf{I}_{2l} \qquad \text{(S1)}$$

where β, $\mathbf{B}_0$, $\mathbf{g}_i$, $\mathbf{S}_i$, $\mathbf{D}$, J, $\mathbf{A}_{ij}$, and $\mathbf{I}_{ij}$ are the Bohr magneton, the external magnetic field vector, the **g** tensor of radical *i*, the electron spin operator of radical *i*, the dipolar coupling tensor, the strength of the isotropic exchange interaction, the hyperfine coupling tensor between nucleus *j* and radical *i*, and the nuclear spin operator of nucleus *j* in radical *i*, respectively. In transient EPR studies, a microwave term has to be added to the spin Hamiltonian of eq S1. It is important to note that pseudo-secular terms of the electron spin-spin interactions are explicitly considered in the analysis. These terms are essential for the modeling of zero-quantum electron coherences and thus provide the basis for the present study.

Formation of quantum oscillations. Generally, the formation of quantum oscillations can be rationalized in terms of the non-adiabatic change of the spin Hamiltonian at the instant of the laser pulse. At time zero, the radical pair is created in a singlet state as a consequence of spin conservation in an ultrafast photochemical reaction. Since the singlet is not an eigenstate of the corresponding spin Hamiltonian, the radical pair starts out in a coherent superposition of eigenstates, which manifests itself as quantum oscillations in an EPR experiment with adequate time resolution.[1-6]

Neglecting all anisotropic hyperfine interactions, the (rotating frame) spin Hamiltonian of the radical pair can be diagonalized analytically for any number of hyperfine coupled nuclei in the donor and acceptor radical ions. Under these conditions, the model predicts the formation of zero-quantum electron coherences whose frequency $\omega_{ZQ}(\Omega)$ sensitively depends on the orientation Ω of the radical pair in the laboratory frame $\mathbf{x}, \mathbf{y}, \mathbf{z}$. Generally, $\omega_{ZQ}(\Omega)$ can be written as[5,6,10-18]

$$\omega_{ZQ}(\Omega) = (1/\hbar)\left\{\left[\tfrac{2}{3}D^{zz}(\Omega) - 2J\right]^2 + \left[\left(g_1^{zz}(\Omega) - g_2^{zz}(\Omega)\right)\beta B_0 + \sum_k a_{1k}M_{1k}^m - \sum_l a_{2l}M_{2l}^n\right]^2\right\}^{1/2}$$
$$M_{1k}^m = I_{1k},\ I_{1k}-1,\ ....\ ,\ -I_{1k} \qquad \text{(S2)}$$
$$M_{2l}^n = I_{2l},\ I_{2l}-1,\ ....\ ,\ -I_{2l}$$

where $D^{zz}(\Omega)$, $g_i^{zz}(\Omega)$, and a_{ij} are the *zz* component of the dipolar coupling tensor, the *zz* component of the **g** tensor of radical *i* and the isotropic hyperfine coupling between nucleus *j* and radical *i*. Inspection of eq S2 reveals that the frequency of the zero-quantum electron oscillations is determined by the electron spin-spin interactions of the radical pair and the difference in the Zeeman and hyperfine interactions of the constituent radicals. For $P_{700}^{\bullet+}A_{1A}^{\bullet-}$ in W-band EPR studies, the Zeeman term provides by far the largest contribution.

In deriving eq S2, all anisotropic hyperfine interactions have been ignored. As a result, light-induced single-quantum nuclear coherences[19] are neglected. The latter is certainly a good approximation for W-band studies, since the amplitude of these coherences strongly decreases with the applied magnetic field. For the analysis of X-band (9.5 GHz) experiments, however, consideration of anisotropic hyperfine interactions is essential.[16,17,19,20]

Radical pair geometry. The orientation dependence of the magnetic tensor elements $g_i^{zz}(\Omega)$ and $D^{zz}(\Omega)$ can be evaluated by a two-fold transformation. In the first step, we transform from the principal axis system $\mathbf{X}_i, \mathbf{Y}_i, \mathbf{Z}_i$ of the respective magnetic tensor to a magnetic reference system, $\mathbf{X}, \mathbf{Y}, \mathbf{Z}$, using the Euler angles[21] $\Omega_i = (\Phi_i, \Theta_i, \Psi_i)$, i = 1A, DA (Figure S1). For convenience, this reference system is chosen parallel to the principal axis system of the **g** tensor of $A_{1A}^{\bullet-}$. In the second

step, we transform by the Euler angles[21] $\Omega = (\Phi, \Theta, 0)$ into the laboratory frame $\mathbf{x}, \mathbf{y}, \mathbf{z}$ (Figure S1). A random distribution of the radical pair with respect to the laboratory frame is considered by averaging over Φ and Θ. In the present study, the orientation of the magnetic tensors (**g** tensor of $P_{700}^{\bullet+}$, dipolar tensor) with respect to the magnetic reference system (**g** tensor of $A_{1A}^{\bullet-}$) is described by the five Euler angles $\Phi_{1A}, \Theta_{1A}, \Psi_{1A}, \Theta_{DA}$ and Ψ_{DA}. They constitute the radical pair geometry of $P_{700}^{\bullet+}A_{1A}^{\bullet-}$. Values for these angles are extracted from a two-dimensional transient W-band experiment using the pronounced variation of the zero-quantum electron oscillations across the powder spectrum.[10-18]

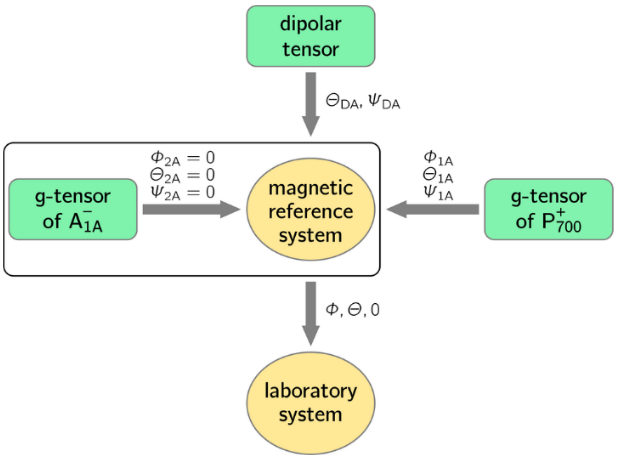

Figure S1. Notation for magnetic tensor systems and Euler transformations used in the EPR model. $P_{700}^{\bullet+}$ is the oxidized primary electron donor. $A_{1A}^{\bullet-}$ is the reduced A-side quinone acceptor. The orientation dependence of the magnetic tensor elements is evaluated by a two-fold transformation. In the first step, we transform from the principal axis system $\mathbf{X}_i, \mathbf{Y}_i, \mathbf{Z}_i$ of the respective magnetic tensor to a magnetic reference system $\mathbf{X}, \mathbf{Y}, \mathbf{Z}$ (**g** tensor of $A_{1A}^{\bullet-}$). In the second step, we transform from $\mathbf{X}, \mathbf{Y}, \mathbf{Z}$ to the laboratory frame $\mathbf{x}, \mathbf{y}, \mathbf{z}$. The orientation of the magnetic tensors (**g** tensor of $P_{700}^{\bullet+}$, dipolar tensor) with respect to the magnetic reference system is described by the five Euler angles[21] $\Phi_{1A}, \Theta_{1A}, \Psi_{1A}, \Theta_{DA}$ and Ψ_{DA} which constitute the radical pair geometry.

2 Bidirectional electron transfer in PSI

Figure S2 depicts the arrangement of the electron transfer cofactors in the cyanobacterium *S. elongatus,* evaluated from an electron density map of PSI at 2.5 Å resolution.[22] One sees that the cofactors are organized in two parallel branches (A, B) relative to a pseudo-C_2 symmetry axis that is perpendicular to the membrane plane. There is now general agreement that both cofactor branches of PSI are competent in electron transfer.[18,23-34] Previous EPR studies reveal a breaking of the approximate C_2 symmetry of the electron transfer cofactors in the electronic structure of $P_{700}^{\bullet+}$.[10,35]. As a result, the EPR signatures of $P_{700}^{\bullet+}A_{1A}^{\bullet-}$ and $P_{700}^{\bullet+}A_{1B}^{\bullet-}$ are markedly different. Thus, in general, quantum oscillations from two distinct radical pairs with different geometries have to be considered in the analysis of a two-dimensional transient nutation experiment.[18] However, pulsed W- and D-band (130 GHz) EPR studies reveal that in wildtype cells of cyanobacteria only the A-side radical pair $P_{700}^{\bullet+}A_{1A}^{\bullet-}$ can be observed *at low temperature.*[31,33,34]

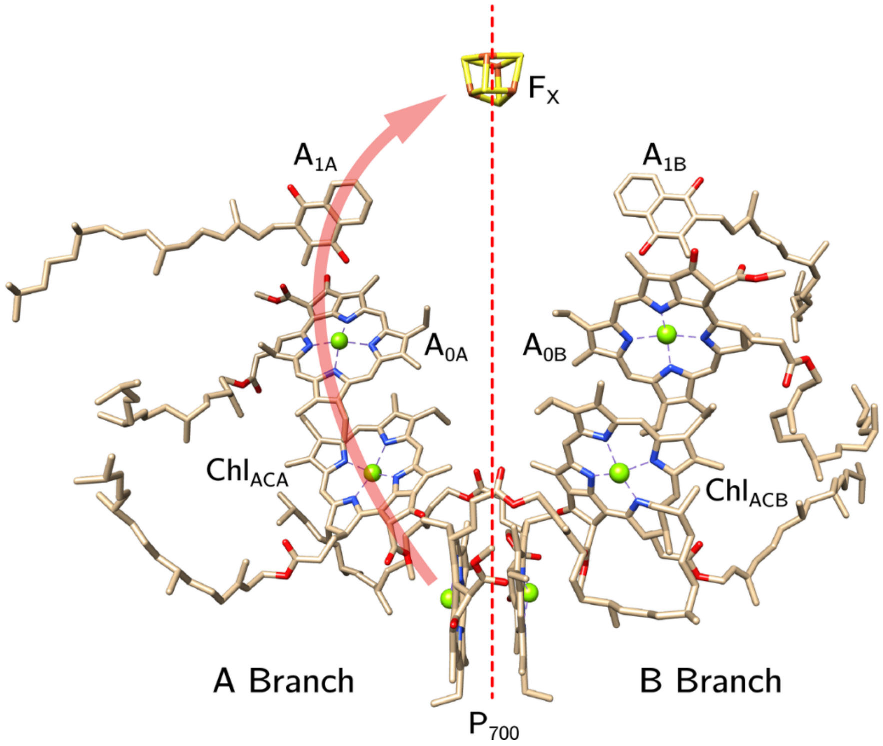

Figure S2. Arrangement of the electron transfer cofactors in PSI taken from an X-ray structure at 2.5 Å resolution.[22] The figure was generated with UCSF Chimera[36] using the crystallographic coordinates deposited at Brookhaven data bank under accession number 1JB0, 2001. The direction view is parallel to the membrane plane. One sees that the electron transfer cofactors are organized in two parallel branches denoted as A- and B-branches. The dashed red line indicates the approximate C_2 symmetry axis collinear with the membrane normal. P_{700} is the primary donor. Chl_{ACA} and Chl_{ACB} are the accessory chlorophylls. A_{0A} and A_{0B} are the chlorophyll acceptors. A_{1A} and A_{1B} are the phylloquinone acceptors. F_x is the first iron-sulfur cluster. The bent arrow indicates the electron transfer pathway observed for wildtype cells of cyanobacteria *at low temperature*.[31,33,34]

3 Magnetic parameters of $P_{700}^{\bullet+}A_{1A}^{\bullet-}$

The fixed magnetic parameters of $P_{700}^{\bullet+}A_{1A}^{\bullet-}$ in PSI, underlying the analysis of the two-dimensional W-band data set, are summarized in Table S1. The **g** tensor components of $P_{700}^{\bullet+}$ and $A_{1A}^{\bullet-}$ have been determined by a number of groups under different experimental conditions and thus vary moderately.[31,37-39] The quoted values were adopted from a pulsed W-band EPR study of $P_{700}^{\bullet+}A_{1A}^{\bullet-}$ in deuterated wildtype cells of *Synechocystis* sp. PCC 6803 at 120 K.[31] The spin-spin coupling parameters of $P_{700}^{\bullet+}A_{1A}^{\bullet-}$, D_A, E_A and J_A, are based on out-of-phase electron spin echo envelope modulation (OOP ESEEM) studies of $P_{700}^{\bullet+}A_{1A}^{\bullet-}$ in non-deuterated wildtype cells of *Chlamydomonas reinhardtii*.[40] Hyperfine interactions in the radical pair were approximated by considering five equivalent ^{14}N nuclei (a_N = 0.060 mT) in $P_{700}^{\bullet+}$ and four equivalent ^{2}H nuclei (a_D = 0.055 mT) in $A_{1A}^{\bullet-}$. This corresponds to second moments of $\langle B_0^2 \rangle = 1.19\times10^{-2}$ mT^2 and $\langle B_0^2 \rangle = 8.1\times10^{-3}$ mT^2 in agreement with published hyperfine parameters for $P_{700}^{\bullet+}$ [20,41] and $A_{1A}^{\bullet-}$.[42,43]

Table S1. Magnetic parameters used in the analysis of a two-dimensional W-band data set[a] of the A-side radical pair $P_{700}^{\bullet+}A_{1A}^{\bullet-}$ in PSI

**g** tensor components[b]		spin-spin coupling[c]	hyperfine interactions[d] line broadening[e] spin relaxation[f]	
$P_{700}^{\bullet+}$	$A_{1A}^{\bullet-}$		$P_{700}^{\bullet+}$	$A_{1A}^{\bullet-}$
g_1^X	g_2^X	D_A	five	four
2.00309	2.00622	-0.171 mT	^{14}N nuclei	^{2}H nuclei
g_1^Y	g_2^Y	E_A	a_N	a_D
2.00255	2.00507	0 mT	0.060 mT	0.055 mT
g_1^Z	g_2^Z	J_A		
2.00227	2.00218	0.0005 mT		

[a]Microwave frequency $\omega/(2\pi) = 94.055$ GHz, microwave field strength $B_1 = 0.04$ mT. [b]Data from a W-band EPR study of $P_{700}^{\bullet+}A_{1A}^{\bullet-}$ in deuterated wildtype cells of *Synechocystis* sp. PCC 6803.[31] [c]Parameter values from OOP ESEEM studies of $P_{700}^{\bullet+}A_{1A}^{\bullet-}$ in non-deuterated wildtype cells of *Chlamydomonas reinhardtii*.[40] [d]Isotropic hyperfine interactions consistent with published hyperfine parameters for $P_{700}^{\bullet+}$ [20,41] and $A_{1A}^{\bullet-}$.[42,43] [e]Inhomogeneous broadening is considered by convolution with a Gaussian of linewidth ΔB_0 = 0.30 mT (fwhm). [f]Electron spin relaxation is taken into account by the transverse relaxation time $T_2 = 1.3$ µs. To conform to the experiment, a resonator bandwidth of $\Delta\nu = 200$ MHz (fwhm) is considered in the calculations.

Inhomogeneous broadening was considered by convolution with a Gaussian of linewidth $\Delta B_0 = 0.30$ mT (fwhm). Electron spin relaxation was taken into account by multiplying each time profile by an exponential decay curve characterized by the transverse relaxation time $T_2 = 1.3$ µs. To conform to the experiment, a resonator bandwidth of $\Delta\nu = 200$ MHz (fwhm) was considered in the calculations. However, in the global fit, a resonator bandwidth of $\Delta\nu = 400$ MHz (fwhm) was employed. Calculations have shown that clearly pronounced modulations of the quantum oscillations improve both the accuracy and the convergence of the fit.

4 Degeneracy of the radical pair geometry

The fit of the two-dimensional W-band EPR data set does not provide a unique set of Euler angles for the geometry $\Phi_{1A}, \Theta_{1A}, \Psi_{1A}, \Theta_{DA}$ and Ψ_{DA} of the radical pair $P_{700}^{\bullet+}A_{1A}^{\bullet-}$. This degeneracy is a general problem in the analysis of magnetic resonance experiments since these techniques cannot in general distinguish between a positive and a negative magnetic axis orientation. In fact, the two **g** tensors exhibit D_{2h} symmetry and are therefore invariant under 180° rotations about their principal axes. The axially symmetric dipolar tensor transforms according to the point group $D_{\infty h}$. Consequently, it is invariant under 180° rotations about any axis perpendicular to the symmetry axis. Applying these magnetic tensor rotations, 32 equivalent radical pair geometries are obtained.[15,18] They are summarized in Table S2.

The 32 degenerate radical pair geometries can be divided into eight groups with four geometries where the latter yield indistinguishable spatial structures. Thus, only an *eight-fold degeneracy* of the radical pair geometry actually exists. For studies of the radical pair spectra, this degeneracy is not critical since all eight representations yield the same spin polarized EPR spectrum. For structural studies, however, the degeneracy of the radical pair geometry has to be eliminated.

This can be achieved by a critical evaluation of the radical pair structures calculated from the eight degenerate W-band geometries and the four possible **g** tensor orientations of $P_{700}^{\bullet+}$ in the A-side radical pair $P_{700}^{\bullet+}A_{1A}^{\bullet-}$ of PSI (Table S3). The crucial parameter is the position of $A_{1A}^{\bullet-}$ in the photosynthetic membrane. It is assumed that a shift of $A_{1A}^{\bullet-}$ relative to the position of A_{1A} in the X-ray structure beyond the EPR uncertainty of ±5.5 Å is unlikely to occur. Using the quinone position as selection criterion, one can eliminate seven of the eight W-band geometries and three of the four **g** tensor orientations. The result is a unique set of Euler angles specifying the cofactor arrangement of $P_{700}^{\bullet+}A_{1A}^{\bullet-}$ in PSI.

Table S2. Equivalent representations of the geometry of the A-side radical pair $P_{700}^{\bullet+}A_{1A}^{\bullet-}$ in PSI

number	$\mathbf{g}_1$	$\mathbf{g}_2$	**D**	orientation of $\mathbf{g}_1$			orientation of **D**	
1	*i*	*i*	*i*	Φ_{1A}	Θ_{1A}	Ψ_{1A}	Θ_{DA}	Ψ_{DA}
2	*X*	*i*	*i*	$2\pi-\Phi_{1A}$	$\pi-\Theta_{1A}$	$\pi+\Psi_{1A}$	Θ_{DA}	Ψ_{DA}
3	*Y*	*i*	*i*	$\pi-\Phi_{1A}$	$\pi-\Theta_{1A}$	$\pi+\Psi_{1A}$	Θ_{DA}	Ψ_{DA}
4	*Z*	*i*	*i*	$\pi+\Phi_{1A}$	Θ_{1A}	Ψ_{1A}	Θ_{DA}	Ψ_{DA}
5	*i*	*X*	*i*	$\pi+\Phi_{1A}$	$\pi-\Theta_{1A}$	$2\pi-\Psi_{1A}$	$\pi-\Theta_{DA}$	$2\pi-\Psi_{DA}$
6	*X*	*X*	*i*	$\pi-\Phi_{1A}$	Θ_{1A}	$\pi-\Psi_{1A}$	$\pi-\Theta_{DA}$	$2\pi-\Psi_{DA}$
7	*Y*	*X*	*i*	$2\pi-\Phi_{1A}$	Θ_{1A}	$\pi-\Psi_{1A}$	$\pi-\Theta_{DA}$	$2\pi-\Psi_{DA}$
8	*Z*	*X*	*i*	Φ_{1A}	$\pi-\Theta_{1A}$	$2\pi-\Psi_{1A}$	$\pi-\Theta_{DA}$	$2\pi-\Psi_{DA}$
9	*i*	*Y*	*i*	$\pi+\Phi_{1A}$	$\pi-\Theta_{1A}$	$\pi-\Psi_{1A}$	$\pi-\Theta_{DA}$	$\pi-\Psi_{DA}$
10	*X*	*Y*	*i*	$\pi-\Phi_{1A}$	Θ_{1A}	$2\pi-\Psi_{1A}$	$\pi-\Theta_{DA}$	$\pi-\Psi_{DA}$
11	*Y*	*Y*	*i*	$2\pi-\Phi_{1A}$	Θ_{1A}	$2\pi-\Psi_{1A}$	$\pi-\Theta_{DA}$	$\pi-\Psi_{DA}$
12	*Z*	*Y*	*i*	Φ_{1A}	$\pi-\Theta_{1A}$	$\pi-\Psi_{1A}$	$\pi-\Theta_{DA}$	$\pi-\Psi_{DA}$
13	*i*	*Z*	*i*	Φ_{1A}	Θ_{1A}	$\pi+\Psi_{1A}$	Θ_{DA}	$\pi+\Psi_{DA}$
14	*X*	*Z*	*i*	$2\pi-\Phi_{1A}$	$\pi-\Theta_{1A}$	Ψ_{1A}	Θ_{DA}	$\pi+\Psi_{DA}$
15	*Y*	*Z*	*i*	$\pi-\Phi_{1A}$	$\pi-\Theta_{1A}$	Ψ_{1A}	Θ_{DA}	$\pi+\Psi_{DA}$
16	*Z*	*Z*	*i*	$\pi+\Phi_{1A}$	Θ_{1A}	$\pi+\Psi_{1A}$	Θ_{DA}	$\pi+\Psi_{DA}$
17	*i*	*i*	*X*	Φ_{1A}	Θ_{1A}	Ψ_{1A}	$\pi-\Theta_{DA}$	$\pi+\Psi_{DA}$
18	*X*	*i*	*X*	$2\pi-\Phi_{1A}$	$\pi-\Theta_{1A}$	$\pi+\Psi_{1A}$	$\pi-\Theta_{DA}$	$\pi+\Psi_{DA}$
19	*Y*	*i*	*X*	$\pi-\Phi_{1A}$	$\pi-\Theta_{1A}$	$\pi+\Psi_{1A}$	$\pi-\Theta_{DA}$	$\pi+\Psi_{DA}$
20	*Z*	*i*	*X*	$\pi+\Phi_{1A}$	Θ_{1A}	Ψ_{1A}	$\pi-\Theta_{DA}$	$\pi+\Psi_{DA}$
21	*i*	*X*	*X*	$\pi+\Phi_{1A}$	$\pi-\Theta_{1A}$	$2\pi-\Psi_{1A}$	Θ_{DA}	$\pi-\Psi_{DA}$
22	*X*	*X*	*X*	$\pi-\Phi_{1A}$	Θ_{1A}	$\pi-\Psi_{1A}$	Θ_{DA}	$\pi-\Psi_{DA}$
23	*Y*	*X*	*X*	$2\pi-\Phi_{1A}$	Θ_{1A}	$\pi-\Psi_{1A}$	Θ_{DA}	$\pi-\Psi_{DA}$
24	*Z*	*X*	*X*	Φ_{1A}	$\pi-\Theta_{1A}$	$2\pi-\Psi_{1A}$	Θ_{DA}	$\pi-\Psi_{DA}$
25	*i*	*Y*	*X*	$\pi+\Phi_{1A}$	$\pi-\Theta_{1A}$	$\pi-\Psi_{1A}$	Θ_{DA}	$2\pi-\Psi_{DA}$
26	*X*	*Y*	*X*	$\pi-\Phi_{1A}$	Θ_{1A}	$2\pi-\Psi_{1A}$	Θ_{DA}	$2\pi-\Psi_{DA}$
27	*Y*	*Y*	*X*	$2\pi-\Phi_{1A}$	Θ_{1A}	$2\pi-\Psi_{1A}$	Θ_{DA}	$2\pi-\Psi_{DA}$
28	*Z*	*Y*	*X*	Φ_{1A}	$\pi-\Theta_{1A}$	$\pi-\Psi_{1A}$	Θ_{DA}	$2\pi-\Psi_{DA}$
29	*i*	*Z*	*X*	Φ_{1A}	Θ_{1A}	$\pi+\Psi_{1A}$	$\pi-\Theta_{DA}$	Ψ_{DA}
30	*X*	*Z*	*X*	$2\pi-\Phi_{1A}$	$\pi-\Theta_{1A}$	Ψ_{1A}	$\pi-\Theta_{DA}$	Ψ_{DA}
31	*Y*	*Z*	*X*	$\pi-\Phi_{1A}$	$\pi-\Theta_{1A}$	Ψ_{1A}	$\pi-\Theta_{DA}$	Ψ_{DA}
32	*Z*	*Z*	*X*	$\pi+\Phi_{1A}$	Θ_{1A}	$\pi+\Psi_{1A}$	$\pi-\Theta_{DA}$	Ψ_{DA}

$\mathbf{g}_1$ and $\mathbf{g}_2$ are the **g** tensors of $P_{700}^{\bullet+}$ and $A_{1A}^{\bullet-}$ (magnetic reference system). **D** is the electron dipolar tensor. *i* denotes the identity operation. *X, Y* and *Z* indicate 180° rotations about one of the principal axes of the magnetic tensor.

5 g Tensor orientation of $P_{700}^{\bullet+}$ in $P_{700}^{\bullet+}A_{1A}^{\bullet-}$

The orientation of the **g** tensor of $P_{700}^{\bullet+}$ in the A-side radical pair $P_{700}^{\bullet+}A_{1A}^{\bullet-}$ of PSI has been determined by high-time resolution X- and W-band EPR using deuterated and ^{15}N-substituted cyanobacteria.[10] To describe this **g** tensor orientation, we define a chlorophyll-based reference system, $\mathbf{X}^{Chl}$, $\mathbf{Y}^{Chl}$, $\mathbf{Z}^{Chl}$, which corresponds to the reference system used for the primary donor in purple bacteria.[44] The Z^{Chl} axis is the chlorophyll normal pointing in the direction of the electron transfer; the Y^{Chl} axis is the projection of the membrane normal onto the chlorophyll plane in opposite direction of the electron transfer. The X^{Chl} axis then lies in the chlorophyll plane perpendicular to $\mathbf{Y}^{Chl}$. Using this reference system, the orientation of the **g** tensor of $P_{700}^{\bullet+}$ in the A-side radical pair can be described by four equivalent sets of Euler angles, denoted by orientation I, II, III and IV (Table S3).[18] Analysis reveals that **g** orientation I is correct.[18]

Table S3. Euler angles characterizing the four possible **g** tensor orientations of $P_{700}^{\bullet+}$ in the A-side radical pair $P_{700}^{\bullet+}A_{1A}^{\bullet-}$ of PSI

	angular values^{a}			
notation^{b}	orientation I	orientation II	orientation III	orientation IV
Φ_1^{Chl}	183°	3°	183°	3°
Θ_1^{Chl}	29°	151°	29°	151°
Ψ_1^{Chl}	220°	320°	40°	140°

^{a}Data from high-time resolution X- and W-band EPR studies on deuterated and ^{15}N-substituted cyanobacteria.[10,18]
^{b}The Euler angles Φ_1^{Chl}, Θ_1^{Chl} and Ψ_1^{Chl} transform from the principal axis system of the **g** tensor of $P_{700}^{\bullet+}$ to the chlorophyll based reference system $\mathbf{X}^{Chl}$, $\mathbf{Y}^{Chl}$, $\mathbf{Z}^{Chl}$.

6 Transient Q-band EPR data of $P_{865}^{\bullet+}Q_A^{\bullet-}$

In Figure S3, we depict transient Q-band EPR spectra and time profiles of the radical pair $P_{865}^{\bullet+}Q_A^{\bullet-}$ in purple bacterial reaction centers (RCs). All experimental and calculated EPR data are reproduced from ref.[15] Copyright 2007 American Chemical Society.

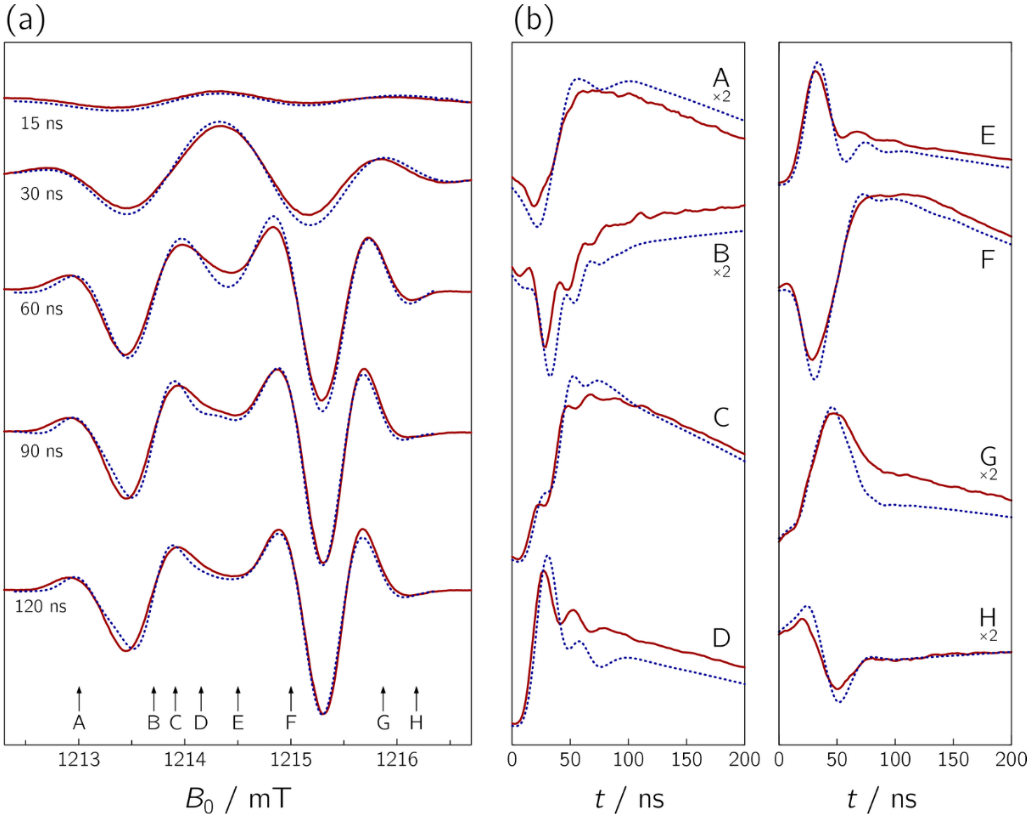

Figure S3. Experimental (full red lines) and calculated (dotted blue lines) transient Q-band EPR data of the radical pair $P_{865}^{\bullet+}Q_A^{\bullet-}$ in RCs of purple bacteria. Positive signals indicate absorptive and negative emissive spin polarization. Microwave frequency $\omega/(2\pi) = 34.064$ GHz. Microwave field strength $B_1 = 0.04$ mT. Experimental data from fully deuterated Fe-removed / Zn-substituted RCs of the purple bacterium *Rhodobacter sphaeroides* at $T = 70$ K. Calculated data using the magnetic and structural parameters in Table S4 (Section 8 in SI). (a) Transient Q-band EPR spectra at five different times after the laser pulse. (b) Time evolution of the transverse Q-band magnetization measured at eight selected field positions (A-H, Figure S3a). There are fast initial oscillations which disappear about 100 ns after the laser pulse. Basically these oscillations represent quantum beats associated with the spin-correlated generation of $P_{865}^{\bullet+}Q_A^{\bullet-}$.

7 Fe-removed / Zn-substituted purple bacterial reaction centers

In native purple bacterial RCs, high-spin Fe^{2+} is magnetically coupled to $Q_A^{\bullet-}$ forming the iron-quinone complex $(Fe^{2+}Q_A^{\bullet-})$,[45] which hampers time-resolved EPR studies of the primary events in purple bacterial photosynthesis. Therefore, this nonheme Fe^{2+} is commonly either removed or removed *and* replaced by diamagnetic Zn^{2+}.[46] In our Q-band EPR study,[15] the latter was achieved by chaotropic treatment using the procedure of Utschig *et al.*[47] The electron transfer rate from the intermediate acceptor $\Phi_A^{\bullet-}$ to Q_A was measured to be $(200\ \text{ps})^{-1}$ (295 K) for the Fe-removed / Zn-substituted samples.[47] This value is in good agreement with electron transfer rates reported for native bacterial RCs.[48]

8 Magnetic and structural parameters of $P_{865}^{\bullet+}Q_A^{\bullet-}$

The magnetic parameters, used in the calculation of the two-dimensional Q-band (34 GHz) data sets of $P_{865}^{\bullet+}Q_A^{\bullet-}$, are summarized in the columns 1-5 of Table S4. The quoted values for the **g** tensor of $P_{865}^{\bullet+}$ and $Q_A^{\bullet-}$ were adopted from a W-band EPR study of the two radical ions in frozen solution under identical conditions.[49] The listed spin-spin coupling parameters are based on an OOP ESEEM study of $P_{865}^{\bullet+}Q_A^{\bullet-}$.[50] Hyperfine interactions in the radical pair were approximated by considering five equivalent ^{14}N nuclei (a_N = 0.0838 mT) in $P_{865}^{\bullet+}$ and four equivalent ^{2}H nuclei (a_D = 0.0411 mT) in $Q_A^{\bullet-}$. This corresponds to second moments of $\langle B_0^2 \rangle = 23.4\times10^{-3}$ mT^2 and $\langle B_0^2 \rangle = 4.5\times10^{-3}$ mT^2 in agreement with the published hyperfine parameters for $P_{865}^{\bullet+}$ and $Q_A^{\bullet-}$ [51-53]. Inhomogeneous broadening was considered by convolution with a Gaussian of linewidth ΔB_0 = 0.29 mT (fwhm). Electron spin relaxation was taken into account by multiplying each time profile by an exponential decay curve characterized by the transverse relaxation time $T_2 = 1.0\ \mu s$. A resonator bandwidth of $\Delta\nu = 110$ MHz (fwhm) was considered by using a Gaussian response function.

Table S4. Magnetic and structural parameters used in the calculation of two-dimensional Q-band data sets[a] for the radical pair $P_{865}^{\bullet+}Q_A^{\bullet-}$ in RCs of purple bacteria

**g** tensor components[b]		spin-spin coupling[c]	hyperfine interactions[d] line broadening[e] spin relaxation[f]		EPR geometry[g]	X-ray geometry[h]
$P_{865}^{\bullet+}$	$Q_A^{\bullet-}$		$P_{865}^{\bullet+}$	$Q_A^{\bullet-}$		
g_1^X	g_2^X	D	five	four	Φ_1	Φ_1
2.00330	2.00660	-0.125 mT	^{14}N nuclei	^{2}H nuclei	26°	19°
g_1^Y	g_2^Y	E	a_N	a_D	Θ_1	Θ_1
2.00250	2.00548	0 mT	0.0838 mT	0.0411 mT	114°	120°
g_1^Z	g_2^Z	J			Ψ_1	Ψ_1
2.00210	2.00220	0 mT			73°	16°
					Θ_D	Θ_D
					67°	73°
					Ψ_D	Ψ_D
					122°	68°

[a]Microwave frequency $\omega/(2\pi) = 34.064$ GHz, microwave field strength $B_1 = 0.04$ mT. [b]Data from a W-band EPR study.[49] [c]Parameters from an OOP ESEEM study of $P_{865}^{\bullet+}Q_A^{\bullet-}$.[50] [d]Isotropic hyperfine interactions consistent with published hyperfine parameters for $P_{865}^{\bullet+}$ [51] and $Q_A^{\bullet-}$.[52,53] [e]Inhomogeneous broadening is considered by convolution with a Gaussian of linewidth $\Delta B_0 = 0.29$ mT (fwhm). [f]Electron spin relaxation is taken into account by the transverse relaxation time $T_2 = 1.0$ μs. A resonator bandwidth of $\Delta\nu = 110$ MHz (fwhm) is considered by using a Gaussian response function. [g]Obtained by a global fit of a two-dimensional Q-band EPR data set.[15] [h]Extracted from an X-ray structure of the photosynthetic RC of *Rhodobacter sphaeroides*.[54]

The structural parameters, employed in the calculation of the two-dimensional Q-band data sets of $P_{865}^{\bullet+}Q_A^{\bullet-}$, are listed in the columns six and seven of Table S4. The EPR geometry was obtained by a global fit of a two-dimensional Q-band data set.[15] The X-ray geometry was extracted from an X-ray structure of the photosynthetic RC of *Rhodobacter sphaeroides*.[54]

9 References

1 Salikhov, K. M.; Bock, C. H.; Stehlik, D. *Appl. Magn. Reson*. 1990, **1**, 195-211.

2 Bittl, R.; Kothe, G. *Chem. Phys. Lett.* 1991, **177**, 547-553.

3 Kothe, G.; Weber, S.; Bittl, R.; Ohmes, E.; Thurnauer, M. C.; Norris, J. R. *Chem. Phys. Lett.* 1991, **186**, 474-480.

4 Zwanenburg, G.; Hore, P. J. *Chem. Phys. Lett*. 1993, **203**, 65-74.

5 Kothe, G.; Weber, S.; Ohmes, E.; Thurnauer, M. C.; Norris, J. R. *J. Phys. Chem*. 1994, **98**, 2706 – 2712.

6 Kothe, G.; Weber, S.; Ohmes, E.; Thurnauer, M. C.; Norris, J. R. *J. Am. Chem. Soc*. 1994, **116**, 7729-7734.

7 Bittl, R.; van der Est, A.; Kamlowski, A.; Lubitz, W.; Stehlik, D. *Chem. Phys. Lett*. 1994, **226**, 349-358.

8 Laukenmann, K.; Weber, S.; Kothe, G.; Oesterle, C.; Angerhofer, A.; Wasielewski, M. R.; Svec, W. A.; Norris, J. R. *J. Phys. Chem.* 1995, **99**, 4324-4329.

9 Heinen, U.; Berthold, T.; Kothe, G.; Stavitski, E.; Galili, T.; Levanon, H.; Wiederrecht, G.; Wasielewski, M. R. *J. Phys. Chem. A* 2002, **106**, 1933-1937.

10 Link, G.; Berthold, T.; Bechtold, M.; Weidner, J.-U.; Ohmes, E.; Tang, J.; Poluektov, O.; Utschig, L.; Schlesselman, S. L.; Thurnauer, M. C.; Kothe, G. *J. Am. Chem. Soc*. 2001, **123**, 4211-4222.

11 Thurnauer, M. C.; Poluektov, O. G.; Kothe, G. *In Biological Magnetic Resonance. Very High Frequency ESR/EPR*, Grinberg, O. Y.; Berliner, L. J.; Eds.; Springer Science: New York, 2004; pp 165-206.

12 Link, G.; Heinen, U.; Bethold, T.; Ohmes, E.; Weidner, J.-U.; Kothe, G. *Z. Phys. Chem.* 2004, **218**, 171-191.

13 Link, G.; Poluektov, O. G.; Utschig, L. M.; Lalevée, J.; Yago, T.; Weidner, J.-U.; Thurnauer, M. C.; Kothe, G. *Magn. Reson. Chem*. 2005, **43**, 103-109.

14 Thurnauer, M. C.; Poluektov, O. G.; Kothe, G. In *Photosystem I: The Light-Driven Plastocyanin: Ferredoxin Oxidoreductase*, Golbeck, J. H., Ed.; Springer: Dordrecht, 2006; pp 339-360.

15 Heinen, U.; Utschig, L. M.; Poluektov, O. G.; Link, G.; Ohmes, E.; Kothe, G. *J. Am. Chem. Soc*. 2007, **129**, 15935-15946.

16 Kothe, G.; Norris, J. R.; Poluektov, O. G.; Thurnauer, M. C. In *Biophysical Techniques in Photosynthesis II*, Aartsma, T. J.; Matysik, J., Eds.; Springer: Dordrecht, 2008, pp 305-323.

17 Kothe, G.; Thurnauer, M. C. *Photosynth. Res*. 2009, **102**, 349-365.

18 Berthold, T.; Donner von Gromoff, E.; Santabarbara, S.; Stehle, P.; Link, G.; Poluektov, O. G.; Heathcote, P.; Beck, C. F.; Thurnauer, M. C.; Kothe, G. *J. Am. Chem. Soc*. 2012, **134**, 5563-5576.

19 Kothe, G.; Bechtold, M.; Link, G.; Ohmes, E.; Weidner, J.-U. *Chem. Phys. Lett*. 1998, **283**, 51-60.

20 Poluektov, O. G.; Utschig, L. M.; Thurnauer, M. C.; Kothe, G. *Appl. Magn. Reson*. 2007, **31**, 123-143.

21 Edmonds, A. R. *Angular Momentum in Quantum Mechanics*; Princeton University Press: Princeton, NJ, 1974; pp 6-8.

22 Jordan, P.; Fromme, P.; Witt, H. T.; Klukas, O.; Saenger, W.; Krauss, N. *Nature* 2001, **411**, 909-917.

23 Poluektov, O.G.; Paschenko, S. V.; Utschig, L. M.; Lakshmi, K. V.; Thurnauer, M. C. *J. Am. Chem. Soc*. 2005, **127**, 11910-11911.

24 Ali, K.; Santabarbara, S.; Heathcote, P.; Evans, M. C. W.; Purton, S. *Biochim. Biophys. Acta* 2006, **1757**, 1623-1633.

25 Li, Y.; van der Est, A.; Lucas, M. G.; Ramesh, V. M.; Gu, F.; Petrenko, A.; Lin, S.; Webber, A. N.; Rappaport, F.; Redding, K. *Proc. Natl. Acad. Sci. U.S.A*. 2006, **103**, 2144-2149.

26 Srinivasan, A.; Golbeck, J. H. *Biochim. Biophys. Acta, Bioenerg*. 2009, **1787**, 1057-1088.

27 Savitsky, A.; Gopta, O.; Mamedov, M.; Golbeck, J. H.; Tikhonov, A.; Möbius, K.; Semenov, A. *Appl. Magn. Reson*. 2010, **37**, 85-102.

28 Santabarbara, S.; Kuprov, I.; Poluektov, O.; Casal, A.; Russell, C. A.; Purton, S.; Evans, M. C. W. *J. Phys. Chem. B* 2010, **114**, 15158-15171.

29 Niklas, J.; Gopta, O.; Epel, B.; Lubitz, W.; Antonkine, M. L. *Appl. Magn. Reson*. 2010, **38**, 187-203.

30 Mula, S.; Savitsky, A.; Möbius, K.; Lubitz, W.; Golbeck, J. H.; Mamedov, M. D.; Semenov, A.; Yu.; van der Est, A. *Photochem. Photobiol. Sci.* 2012, **11**, 946-956.

31 Savitsky, A.; Niklas, J.; Golbeck, J. H.; Möbius, K.; Lubitz, W. *J. Phys. Chem. B* 2013, **117**, 11184-11199.

32 McConnell, M. D.; Sun, J.; Siavashi, R.; Webber, A.; Redding, K. E.; Golbeck, J. H.; van der Est, A. *Biophys. Biochim. Acta, Bioenerg*. 2015, **1847**, 429-440.

33 Poluektov, O.; Utschig, L.M. *J. Phys. Chem. B* 2015, **119**, 13771-13776.

34 Poluektov, O. G.; Niklas, J.; Utschig, L. M. *J. Phys. Chem. B* 2019, **123**, 7536-7544.

35 Zech, S. G.; Hofbauer, W.; Kamlowski, A.; Fromme, P.; Stehlik, D.; Lubitz, W.; Bittl, R. *J. Phys. Chem. B* 2000, **104**, 9728-9739.

36 Pettersen, E. F.; Goddard, T. D.; Huang, C. C.; Couch, G. S.; Greenblatt, D. M.; Meng, E. C.; Ferrin, T. E. *J. Comput. Chem*. 2004, **25**, 1605-1612.

37 Van der Est, A.; Prisner, T.; Bittl, R.; Fromme, P.; Lubitz, W.; Möbius, K.; Stehlik, D. *J. Phys.*

Chem. B 1997, **101**, 1437-1443.

38 Bratt, P. J.; Rohrer, M.; Krzystek, J.; Evans, M. C. W.; Brunel, L.-C.; Angerhofer, A. *J. Phys. Chem. B* 1997, **101**, 9686-9689.

39 MacMillan, F.; Hanley, J.; van der Weerd, L.; Knüpling, M.; Un, S.; Rutherford, A. W. *Biochemistry* 1997, **36**, 9297-9303.

40 Santabarbara, S.; Kuprov, I.; Fairclough, W. V.; Purton, S.; Hore, P. J.; Heathcote, P.; Evans, M. C. W. *Biochemistry* 2005, **44**, 2119-2128.

41 Käss, H.; Bittersmann-Weidlich, E.; Andréasson, L.-E.; Bönigk, B.; Lubitz, W. *Chem. Phys.* 1995, **194**, 419-432.

42 Righy, S. E. J.; Evans, M. C. W.; Heathcote, P. *Biochemistry* 1996, **35**, 6651-6656.

43 Niklas, J.; Epel, B.; Antonkine, M. L.; Sinnecker, S.; Pandelia, M.-E.; Lubitz, W. *J. Phys. Chem. B* 2009, **113**, 10367-10379.

44 Klette, R.; Törring, J. T.; Plato, M.; Möbius, K.; Bönigk, B.; Lubitz, W. *J. Phys. Chem*. 1993, **97**, 2015-2020.

45 Butler, W. F.; Calvo, R.; Fredkin, D. R.; Isaacson, R. A.; Okamura, M. Y., Feher, G. *Biophys. J.* 1984, **45**, 947-973.

46 Kirmaier, C.; Holton, D.; Debus, R. J.; Feher, G.; Okamura, M. Y. *Proc. Natl. Acad. Sci. U.S.A.* 1986, **83**, 6407-6411.

47 Utschig, L. M.; Greenfield, S. R.; Tang, J.; Laible, P.D.; Thurnauer, M. C. *Biochemistry* 1997**,** **36**, 8548-8558.

48 Kirmaier, C.; Holton, D.; Parson, W. W. *Biochim. Biophys. Acta* 1985, **810**, 33-48.

49 Burghaus, O.; Plato, M.; Rohrer, M.; Möbius, K.; MacMillan F.; Lubitz, W. *J. Phys. Chem.* 1993, **97**, 7639-7647.

50 Bittl, R.; Zech, S. G. *J. Phys. Chem. B* 1997, **101**, 1429-1436.

51 Käss, H.; Rautter, J.; Bönigk, B.; Höfer, P.; Lubitz, W. *J. Phys. Chem*. 1995, **99**, 436-448.

52 Rohrer, M.; MacMillan, F.; Prisner, T. F.; Gardiner, A. T.; Möbius, K.; Lubitz, W. *J. Phys. Chem. B* 1998, **102**, 4648-4647.

53 Flores, M.; Isaa**c**son, R.; Abresch, E.; Calvo, R.; Lubitz, W.; Feher, G. *Biophys. J.* 1998, **90**, 3356-3362.

54 Ermler, U.; Fritzsch, G.; Buchanan, S. K.; Michel, H. *Structure* 1994, **2**, 925-936.

10 Acknowledgments

Molecular graphics were performed with UCSF Chimera, developed by the Resource for Biocomputing, Visualization, and Informatics at the University of California, San Francisco, with support from NIH P41-GM103311.
